# Deciphering the genetic basis of salinity tolerance in a diverse panel of cultivated and wild soybean accessions by genome-wide association mapping

**DOI:** 10.1007/s00122-024-04752-4

**Published:** 2024-09-28

**Authors:** Rajat Pruthi, Chanderkant Chaudhary, Sandeep Chapagain, Mostafa Mohamed Elbasuoni Abozaid, Prabhat Rana, Ravi Kiran Reddy Kondi, Roberto Fritsche-Neto, Prasanta K. Subudhi

**Affiliations:** 1 School of Plant, Environmental, and Soil Sciences, Louisiana State University Agricultural Center, Baton Rouge, LA 70803 USA; 2 LSU AgCenter, H. Rouse Caffey Rice Research Station, Rayne, USA; 3 Botany and Microbiology Department, Faculty of Sciences, Al-Azhar University, Cairo, 11884 Egypt

## Abstract

****Key message**:**

In a genome-wide association study involving 269 cultivated and wild soybean accessions, potential salt tolerance donors were identified along with significant markers and candidate genes, such as *GmKUP6* and *GmWRKY33*.

****Abstract**:**

Salt stress remains a significant challenge in agricultural systems, notably impacting soybean productivity worldwide. A comprehensive genome-wide association study (GWAS) was conducted to elucidate the genetic underpinnings of salt tolerance and identify novel source of salt tolerance among soybean genotypes. A diverse panel comprising 269 wild and cultivated soybean accessions was subjected to saline stress under controlled greenhouse conditions. Phenotypic data revealed that salt tolerance of soybean germplasm accessions was heavily compromised by the accumulation of sodium and chloride, as indicated by highly significant positive correlations of leaf scorching score with leaf sodium/chloride content. The GWAS analysis, leveraging a dataset of 32,832 SNPs, unveiled 32 significant marker-trait associations (MTAs) across seven traits associated with salt tolerance. These markers explained a substantial portion of the phenotypic variation, ranging from 14 to 52%. Notably, 11 markers surpassed Bonferroni’s correction threshold, exhibiting highly significant associations with the respective traits. Gene Ontology enrichment analysis conducted within a 100 Kb range of the identified MTAs highlighted candidate genes such as potassium transporter 6 (*GmKUP6*), cation hydrogen exchanger (*GmCHX15*), and *GmWRKY33*. Expression levels of *GmKUP6* and *GmWRKY33* significantly varied between salt-tolerant and salt-susceptible soybean accessions under salt stress. The genetic markers and candidate genes identified in this study hold promise for developing soybean varieties resilient to salinity stress, thereby mitigating its adverse effects.

**Supplementary Information:**

The online version contains supplementary material available at 10.1007/s00122-024-04752-4.

## Introduction

Soil salinization is a major global issue impacting agricultural productivity and sustainability. Approximately one-fifth of the world's irrigated agricultural land is affected by salinization, leading to significant crop losses estimated at around 27.3 billion USD annually (FAO 2015; Qadir et al. 2014). The problem is exacerbated by natural and human-induced factors, such as native rock weathering, high surface evaporation, low precipitation, poor cultural practices, and seawater intrusions in coastal areas during hurricanes (Shrivastava and Kumar 2015). Projections suggest that over 50% of farmable land will be salinized by 2050 (Stephenson et al. 2010), posing a severe challenge to food security given the global population's projected growth to 9.7 billion by 2050 (UN 2022). Meeting the food requirements of such a large population with the available natural resources is challenging (Godfray et al. 2010). Therefore, efforts should focus on developing high-yielding varieties with enhanced tolerance to abiotic stresses, including salinity, by utilizing the naturally occurring tolerance in existing germplasm (Blumwald and Grover 2006).

Soybean (*Glycine max* L. Merr.; n = 20) is an important cash crop that significantly contributes to the world’s protein meal consumption and oilseed production (Soystats 2022). Although soybeans are classified as moderately tolerant to saline stress with a salinity threshold of 5 dS/m, high-yielding cultivars show severe susceptibility at higher salt concentrations (Ashraf and Wu 1994). Salt buildup in soybeans severely affects various growth phases, including germination, seedling, and reproductive stages (Phang et al. 2008). Parker et al. (1983) observed 37% yield reduction and foliar symptoms such as browning, scorching, and chlorosis in salt-sensitive genotypes. The salt tolerance of soybean cultivars depends on their ability to exclude Cl^−^ from the foliar tissue (Abel 1969). Tolerant cultivars (excluders) tend to accumulate Cl^−^ in stems and roots, avoiding Cl^−^ accumulation in leaves and thus showing minimal leaf scorching (Patil et al. 2016; Zeng et al. 2017). However, recent studies have shown that Na^+^ accumulation in leaves is equally detrimental to soybean growth under saline conditions (Lenis et al. 2011; Do et al. 2016, 2018, 2019; Patil et al. 2016). *Glycine soja* (wild soybean) is sensitive to Na^+^, whereas the cultivated type (*Glycine max*) is sensitive to Cl^−^ (Luo et al. 2005).

Both forward and reverse genetics have been employed to decipher the molecular basis of salt tolerance in soybeans. Quantitative trait locus (QTL) mapping in bi-parental populations is a well-established and powerful approach for genetically dissecting salinity tolerance traits. A major QTL, from the cultivar S-100 (PI 548488), which explained 60% of the phenotypic variation, was identified on chromosome 3 (Lee et al. 2004). This QTL has been validated using populations involving different genotypes in various studies (Guan et al. 2014; Qi et al. 2014; Do et al. 2018). Map-based cloning and whole-genome resequencing led to the cloning of the gene *GmSALT3* (*Glyma03g32900*; Wm82.a1.v1.1) in the QTL region (Guan et al. 2014; Qi et al. 2014), establishing it as a major source of salinity tolerance in soybean germplasm (Zeng et al. 2017; Do et al. 2019).

In recent years, association mapping has emerged as a powerful tool for unraveling the genetic architecture of various plant traits (Zhu et al. 2008; Alqudah et al. 2020). This approach has become widely adopted across diverse crops, including rice (Huang et al. 2010), maize (Buckler et al. 2009), and *Arabidopsis thaliana* (Atwell et al. 2010), due to its advantages over traditional QTL mapping. In soybean, genome-wide association studies (GWAS) have been successfully implemented to discover marker-trait association (MTA) for traits such as cyst nematode resistance (Vuong et al. 2015), resistance to sudden death syndrome (Wen et al. 2015), and seed oil and protein concentration (Bandillo et al. 2015). Three GWAS studies have identified salinity-responsive chromosomal regions in soybean at the seedling stage (Patil et al. 2016; Zeng et al. 2017; Do et al. 2019). Do et al. (2019) conducted GWAS using 305 soybean accessions and identified significant MTAs on chromosomes 1, 3, 8, and 18. Among these, MTAs on chromosome 3 validated the known salt tolerance gene *GmSALT3*. Additionally, Patil et al. (2016) and Zeng et al. (2017) corroborated the role of *GmSALT3* in imparting salt tolerance across soybean cultivars. These studies also identified multiple significant MTAs on different chromosomes, suggesting the involvement of other genes in salinity tolerance in soybean (Zeng et al. 2017; Do et al. 2019).

In this study, we used a panel of 269 genetically diverse soybean accessions to evaluate salinity tolerance under greenhouse conditions. Single Nucleotide Polymorphism (SNP) markers for these accessions were retrieved from the Illumina Infinium SoySNP50K iSelect BeadChip dataset (https://www.soybase.org/) (Song et al. 2013, 2015). Our objectives in this study were to identify novel donors with salt tolerance, uncover new genomic regions associated with salinity tolerance, confirm previously identified chromosomal regions and MTAs associated with salinity tolerance, and validate the markers identified in this study using genomic prediction models.

## Materials and methods

### Plant materials

Two hundred and sixty-nine soybean genetically diverse accessions from different countries were obtained from the USDA Soybean Germplasm Collection. This diverse panel included 214 cultivated soybean accessions (*Glycine max*) and 53 wild accessions (*Glycine soja*). The salt-susceptible genotype ‘Hutcheson’ (PI 518664) and the salt-tolerant genotype ‘Lee’ (PI 548656) were included as checks. According to data from Germplasm Resources Information Network (GRIN), the germplasm accessions originated from 34 countries, with maturity groups ranging from 000 to VII (Table S1).

### Phenotypic evaluation

The soybean panel was evaluated for salinity tolerance at the LSU Agricultural Center Central Research Station greenhouse under ambient temperature (25–28 °C) and light conditions. The entire experiment was conducted in three replications. Each genotype was represented by five plants per replication, grown in conical tubes filled with sterile sand. During the initial planting stage, the plants were irrigated with reverse osmosis (RO) water with pH 7.0. After germination, all the plants were grown in a nutrient solution containing 0.1% Jack's professional fertilizer 20–20–20 (J.R. Peters, Inc., Allentown, PA, USA), and Peters professional liquid S.T.E.M. Supplement (Everris Na Inc. Dublin, OH, USA) (12.5 ml per 10L). When the genotypes reached the V2 growth stage, plants were exposed to 60 mM NaCl solution for three days (Fehr et al. 1971). Then the NaCl concentration was increased to 150 mM and continued until the susceptible check ‘Hutcheson’ showed severe leaf scorching symptoms (Shi et al. 2018). Based on the severity of leaf scorching, each soybean plant was visually scored on a scale from 1 to 5, where ‘1’ represents no apparent chlorosis, ‘2’ indicates slight chlorosis (25% leaves showing chlorosis), ‘3’ denotes moderate chlorosis (50% leaves showing chlorosis), ‘4’ signifies severe chlorosis (75% leaves showing chlorosis), and ‘5’ means all leaves showing chlorosis and withering (Lee et al. 2008). The mean leaf scorching score (LSS) per replication was calculated using a previously described formula (Lee et al. 2008). Leaf chlorophyll content was measured from the second fully expanded leaf from the top 12 days after the salt treatment using a SPAD 502 chlorophyll meter (Spectrum Technologies, Inc. Aurora, IL, USA).

To determine the leaf sodium content (LSC), leaf potassium content (LKC), leaf calcium content (LCaC), and leaf chloride content (LCC), trifoliate leaves were harvested from five seedlings. The collected leaf tissues were oven-dried at 65 °C for 7 days and then ground using a coffee grinder. In brief, 0.5 g of homogenized leaf tissue was digested with 2.2 mL of deionized water and 5 mL of concentrated HNO_3_ at 125 °C for 2 h 45 min. Subsequently, 3 mL of H_2_O_2_ was added, and digestion continued at 125 °C for 45 min (Benton 1991; Jones 2001). After complete digestion, the samples were diluted to a total volume of 20 mL with deionized water. To prevent excessive ion introduction into the plasma, the samples were further diluted to a 5X dilution, resulting in a final dilution factor of 200. These diluted samples and standard solutions were then analyzed using SPECTRO ARCOS Inductively Coupled Plasma – Optical Emission Spectrometry (ICP-OES) to determine the final concentrations of Na^+^, K^+^, Ca^2+^, and Cl^−^.

### Statistical analysis

Statistical Analysis System 9.4 was used to analyze the phenotypic data (SAS Inc, 2012). The PROC CORR function was employed to calculate correlation between various physiological and morphological characteristics. Descriptive statistics for each trait were computed using the PROC UNIVARIATE technique. The mean value of each trait was used for genome-wide association analysis. The following model was implemented to compute the Best Linear Unbiased Predictors (BLUPs) for the genotypes as well as to estimate the variance components and Cullis heritability (Cullis et al. 2006).

$$y = Xb + Zg +\upepsilon$$

where y is the vector of phenotypic value of salt-related trait; b is the replicate fixed effect; *g* is the vector of random genetic effect with $N\left( {0,G\sigma_{g}^{2} } \right)$. G is the genomic relationship matrix (GRM) for the 269 genotypes and ${\sigma }_{g}^{2}$ is the genetic variance; ϵ is the random effect of the residual with $N\left( {0,R\sigma_{e}^{2} } \right)$, where R represents the variance–covariance matrix of the residual effects. X is an incidence matrix associated with a fixed effect and Z is a random effect incidence matrix. The clustering of the genotypes was done using the k-means algorithm (Li and Wu 2012).

### Genotyping dataset and quality control

Genotypic data for 269 soybean germplasm accessions were obtained from Illumina Infinium SoySNP50K Bead Chip data, accessible through the soybean database (http://www.soybase.org). The dataset underwent filtering based on criteria including a minor allele frequency (MAF) threshold of 5%, removal of SNP markers with more than 5% missing data, and the exclusion of markers with linkage disequilibrium (LD) exceeding 0.99 with other markers using the package SNPRelate (Zheng et al. 2023). Subsequently, the filtered marker dataset was utilized for downstream analysis.

### Linkage disequilibrium and population structure

A pairwise LD assessment was conducted on the dataset comprising 269 genetically diverse accessions. This involved calculating the squared correlation coefficient (r^2^) and distances between adjacent SNP loci on each chromosome. The analysis was performed in R, utilizing the "SNPRelate" and "reshape" packages (Wickham 2007; Zheng et al. 2023). For LD decay analysis on a per-chromosome basis, distances were segmented into 10-kilobase (kb) bins, and r^2^ values were computed within each bin. These r^2^ values were then averaged to obtain the mean r^2^ per chromosome. The overall half LD decay was determined as the chromosomal distance at which r^2^ dropped to half its maximum value. To achieve this, an LD matrix was computed in Tassel 5.2 (Bradbury et al. 2007) using the sliding window approach with an LD window size of 2000. The LD decay rate was quantified using the classical r^2^ metric (Hill and Weir 1988) and visualized in R Studio using nonlinear model functions (Remington et al. 2001). Principal Component Analysis (PCA) was conducted on the filtered SNPs dataset in R Studio using ‘PCAtools’ to analyze population structure (Blighe and Lun 2022). The optimal number of Principal Components (PCs) was selected based on assessing the proportion of variance explained by each PC. Additionally, to measure genetic relatedness among genotypes, a kinship matrix was constructed using the VanRaden method and visualized using the ‘heatmap3’ package in R Studio (Zhao et al. 2014; VanRaden 2008).

### Genome-wide association mapping (GWAS)

Genome-wide association mapping for salt-responsive traits was conducted using Genome Association and Prediction Integrated Tool (GAPIT) package (Lipka et al. 2012) in R employing Bayesian Information and Linkage Disequilibrium Iteratively Nested Keyway (BLINK) (Huang et al. 2019). BLINK, an enhanced model, builds upon the Fixed and Random Model Circulating Probability Unification (FarmCPU), offering improved statistical robustness and efficiency in identifying significant SNPs associated with traits of interest. Unlike FarmCPU, which relies on an assumption of an even marker distribution across the genome and consists of both fixed and random effect models, BLINK incorporates LD information.

A significance threshold of −log10(P) ≥ 4 was set to declare significant marker-trait associations (MTAs) for various salt-responsive traits. This threshold was selected to balance stringency with the ability to detect meaningful associations within our dataset. Additionally, to account for multiple testing, adjusted *P*-values were computed using the Bonferroni-correction method alongside the pre-defined significance threshold to identify highly significant markers. Pairwise r^2^ values were computed for 15 upstream and downstream SNPs of significant MTAs identified on chromosomes 1 and 14. The LD heatmap surrounding these peaks was plotted in R using the ‘gaston’ package (Dandine-Roulland and Perdry 2017). The geneHapR package was utilized to construct the ANOVA (Analysis of Variance) framework for evaluating haplotypes across the identified MTAs (Zhang et al. 2023). A comprehensive search was conducted within a 100-kilobase (kb) region around significant marker-trait associations (MTAs) to identify potential candidate genes. Fixed 100 kb distance provides a consistent framework for evaluating associations, though it may not fully capture local LD structures. The identified candidate genes were subsequently annotated using the ‘Glyma.Wm.82.a2 soybean reference genome’ (www.soybase.org).

### Gene ontology (GO) analysis

GO analysis (Ashburner et al. 2000) for candidate genes within the 100 Kb region of significant MTAs was conducted using the BiNGO plugin (Maere et al. 2005) in Cytoscape (Shannon et al. 2003; https://cytoscape.org/). The visual representation of GO-enriched terms was depicted as scatterplots using the REVIGO (reduce + visualize gene ontology) (Supek et al. 2011; http://revigo.irb.hr/) web servers. A significance threshold of *P*-value < 0.05 was applied in this analysis. Based on function, Biological Processes (BP), Cellular Processes (CP), and Molecular Function (MF) were categorized into various clusters, and GO terms involved in the respective clusters were cited in tabular form.

### Total RNA extraction and quantitative real time-PCR (qRT-PCR)

Two salt-tolerant (PI 561363; PI 507692A) and one salt-susceptible (PI 601984) soybean accessions were grown in sand culture with three replications. For both control and stress conditions, three seedlings were grown in each replication, and at the V2 stage, soybean seedlings were subjected to 150 mM salt stress. Leaf samples were collected at 0 h, 6 h, 24 h, and 48 h after imposition of salinity stress. Total RNA was extracted from leaf tissues of three biological replicates using the Zymo Direct-zol RNA Miniprep Kit (Zymo Research, CA, USA) following the manufacturer’s instructions. The quality of the total RNA was evaluated on a 1.2% agarose gel, and the RNA quantity was determined using an ND-1000 spectrophotometer. The cDNA was synthesized using qScript® cDNA Supermix (Quanta BioSciences, Gaithersburg, MD, USA) as per the manufacturer’s protocol.

Candidate genes for qRT-PCR were selected based on GO analysis. Primers were designed using Primer3Plus, with Elongation factor 1 alpha (*EF1α*; *Glyma.02G276600*) serving as the internal control. The qRT-PCR reactions were conducted with two technical replicates obtained from three independent biological replicates on a QuantStudio 3 Real-Time PCR system (Applied Biosystems, Waltham, MA, USA) using PerfeCTa™ SYBR® Green FastMix™, Low ROX™ (Quanta BioSciences, Gaithersburg, MD, USA). The relative change in gene expression levels was determined using the 2^–∆∆CT^ method (Livak and Schmittgen 2001). A two-way factorial ANOVA was conducted to compare the effect of genotypes, time points, and their interaction on the relative gene expression levels. Following the ANOVA, Tukey's post-hoc test was performed for detailed comparisons of gene expression across different genotypes and time points.

### SNP Heritability

To compute the percentage of phenotype variation explained by each significant SNP marker, we adjusted the genomic relationship matrix by excluding the significant markers. Subsequently, regression analysis was conducted, incorporating the genomic relationship matrix (GRM) and the significant SNP markers for the traits as random effects, using the following equation:

$$y^{*} = Z_{1} g + Z_{2} SNP_{i} + \varepsilon$$

where ${y}^{*}$ represents the vector of mean values for a particular trait; g denotes random genotype effect, which follows a normal distribution with $N\left( {0,\;G\sigma_{g}^{2} } \right)$, where $\left( G \right)$ is the additive kinship matrix; $SNP_{i}$ signifies the random effect of the ith SNP with $N\left( {0,\;I\sigma_{SNPi}^{2} } \right)$, where I represents the identity matrix; $\varepsilon$ indicates the random residual effect with $N\left( {0,\;R\sigma_{e}^{2} } \right)$, where R represents variance–covariance matrix of the residual effects; $Z_{1} ,Z_{i + 1} , \ldots ,Z_{p + 1 }$ are the random effect incidence matrices.

From the above equation, we obtained the genotype, SNP, and residual variance, and these variance components were used to calculate narrow sense heritability for each marker using the following formula:

$$h\left( {SNP_{i} } \right) = \frac{{\sigma_{{SNP_{i} }}^{2} }}{{\sigma_{g}^{2} + \sigma_{{SNP_{i} }}^{2} + \sigma_{e}^{2} }}$$

A similar procedure was employed to calculate the total percent phenotypic variation explained by all the significant SNPs for a particular trait. The procedure involved the exclusion of all the significant markers for the trait from the genomic relationship matrix (GRM), followed by multiple regression with the GRM, and all the significant SNP markers were introduced as random effects using the following equation:

$$y^{*} = Z_{1} g + \mathop \sum \limits_{i = 1}^{p} Z_{i + 1} SNPi + \varepsilon$$

Based on the above formula, the narrow sense heritability associated with all the significant markers for a single trait was estimated using the following model:

$$h_{{\left( {total} \right)}} = \frac{{\mathop \sum \nolimits_{i = 1}^{p} \sigma_{{SNP_{i} }}^{2} }}{{\sigma_{g}^{2} + \mathop \sum \nolimits_{i = 1}^{p} \sigma_{{SNP_{i} }}^{2} + \sigma_{e}^{2} }}$$

### Genomic selection

A set of thirty-two significant markers identified in GWAS analysis was used in conjunction with randomly sampled 32 SNP markers for the prediction of Genomic Estimated Breeding Values (GEBV) in R using the ‘Sommer’ package (Covarrubias-Pazaran 2016). The cross-validation process involved dividing the data into training and validation sets using a ‘nfold’ value of 5 and repeated 20 times to ensure the consistency and reliability of the results. The standard GBLUP model, focusing on additive genetic effects, was utilized for prediction purposes.

$$y_{i} = \mu + g_{i} + \varepsilon_{i}$$

where μ represents the overall mean;$g_{i}$ is the random effect of the ith genotype, denoted as $g_{i} \sim {\mathcal{N}}\left( {0,\sigma_{g}^{2} G_{i} } \right)$ with the genomic relationship matrix (G) estimated as G = X * XT/p, where X is the n × p matrix of centered and standardized markers, n is the number of genotypes, p is the number of markers, and $\varepsilon_{i}$ is the residual effects denoted as $\varepsilon_{i} \sim {\mathcal{N}}\left( {0,I\sigma_{\varepsilon }^{2} } \right)$. The predictive capability was assessed by determining the Pearson’s correlation coefficient between the observed and predicted phenotypic values within the test dataset. Marker-based narrow sense heritability $h_{g}^{2}$ for different traits was calculated using the following formula:

$$h_{g}^{2} = \frac{{\sigma_{g}^{2} }}{{\sigma_{g}^{2} + \sigma_{\varepsilon }^{2} }}$$

where ${\sigma }_{g}^{2}$ represents the additive genetic variance calculated using genomic relationship matrix (GRM) and $\sigma_{\varepsilon }^{2}$ is residual error variance.

## Results

### Phenotypic variation, heritability, and trait correlations

**Fig. 1:**
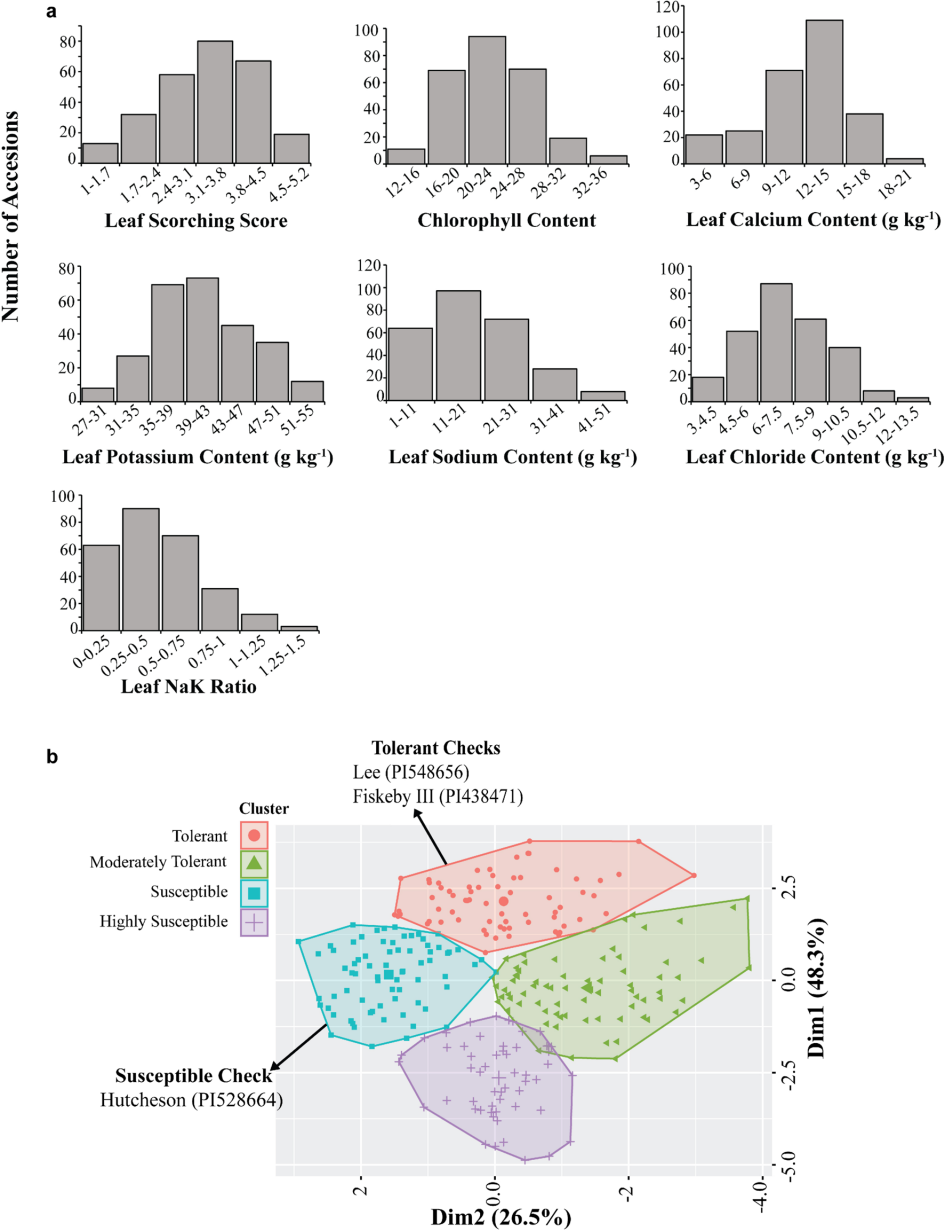
Phenotypic distribution and clustering of the germplasm accessions. **a** Distribution of seven different morphological and physiological traits under saline stress. LSS, leaf scorching score; CC, chlorophyll content measured by SPAD meter; LCaC, leaf calcium content; LPC, leaf potassium content; LSC, leaf sodium content; LCC, leaf chloride content; LNaK; leaf sodium to potassium ratio. **b** Phenotypic clustering of soybean germplasm using the k-means clustering method based on seven different morphological and physiological traits under seedling stage salinity stress

Significant phenotypic variation was observed across all traits, including LSS, CC, LCaC, LPC, LSC, LCC, and LNaK (Table 1). The range of variation was wide for all traits: LSS ranged from 1 to 5, CC from 13.9 to 35.9, LCaC from 4.2 to 11.9 (g kg^−1^), LPC from 27.4 to 54.4 (g kg^−1^), LSC from 2.3 to 49 (g kg^−1^), LCC from 3.3 to 12.7 (g kg^−1^), and LNaK from 0.1 to 1.4 (Table 1; Fig. 1a).

**Table 1:** Means, summary statistics, and Pearson Correlations among salt tolerance traits in a diverse panel of 269 soybean accessions

Pearson's Correlation											
Trait^a^	Min^b^	Max^c^	Mean	h^2d^	LSS	CC	LCaC	LPC	LSC	LCC	LNaK
LSS	1.0	5.0	3.3	84	1.00						
CC	13.9	35.6	22.4	76	−0.74***	1.00					
LCaC	4.2	20.6	11.9	67	−0.01	−0.26***	1.00				
LPC	27.4	54.4	41.2	54	−0.01	−0.22***	0.52***	1.00			
LSC	2.3	49.4	19.3	54	0.63***	−0.32***	−0.14*	−0.24***	1.00		
LCC	3.3	12.7	7.3	44	0.50***	−0.30***	−0.04	0.05	0.75***	1.00	
LNaK	0.1	1.4	0.5	69	0.58***	−0.26***	−0.24***	−0.44***	0.97***	0.66***	1.00

^a^LSS, leaf scorching score; CC, chlorophyll content measured by the SPAD meter; LCaC, leaf calcium content; LPC, leaf potassium content;

LSC, leaf sodium content; LCC, leaf chloride content; LNaK; leaf sodium to potassium ratio

^b^Min, minimum; ^c^Max, maximum; ^**d**^ h2, Cullis^’^s heritability (%)

*significant at 0.05 probability level; **significant at 0.01 probability level; ***significant at 0.001 probability level

Pearson correlation coefficients and Cullis’s heritability values were calculated for the seven different salt-related traits (Table 1). A negative and significant correlation was observed between LSS and CC (− 0.74), while correlations were significantly positive between LSS and LSC (0.63), LCC (0.50), and LNaK (0.58). Additionally, a significant positive correlation was found between LCaC and LPC (0.52). Furthermore, positive correlations were observed among LSC, LCC, and LNaK, whereas all these traits exhibited negative correlations with CC and LCaC. This suggests that susceptibility in the germplasm panel was attributed to excessive accumulation of Na^+^ and Cl^−^. The heritability estimates varied among the traits, with the lowest value being 44% for LCC. In contrast, LSS, CC, LCaC, LPC, LSC, and LNaK exhibited higher heritability values of 85%, 76%, 67%, 54%, 54%, and 69%, respectively.

### Phenotypic clustering

The analysis of phenotypic responses to saline stress within the germplasm panel revealed the clustering of germplasm accessions into four distinct groups (Table 2; Fig. 1b). Cluster 1 emerged as the salt-tolerant group, exhibiting the lowest mean LSS (2.3), a high mean CC (25.2), and lower accumulation of Na^+^ and Cl^−^ in the plant leaves (Mean LSC: 9.4 g kg^−1^, Mean LCC: 5.4 g kg^−1^, Mean LNaK: 0.2). Notably, this cluster included the established salt-tolerant cultivar Lee (PI548656) alongside the previously identified salt-tolerant genotype Fiskeby III (PI438471).

**Table 2:** The average value of each cluster identified through k-means cluster analysis. Various morphological and physiological traits under salt stress in a diverse panel of soybean accessions were used for analysis

Cluster^a^	Mean**@**LSS^b^	Mean**@**CC	Mean**@**LCaC	Mean**@**LPC	Mean**@**LSC	Mean**@**LCC	Mean**@**LNaK
(Tolerant)	2.3	25.2	13.1	41.7	9.4	5.4	0.2
(Moderately Tolerant)	3.2	24.5	8.5	37.0	20.5	7.4	0.6
(Susceptible)	3.7	19.3	14.2	46.7	16.0	7.4	0.3
(Highly Susceptible)	4.2	19.9	12.1	38.8	34.2	9.2	0.9

^a^Four different clusters identified by k-means cluster analysis

^b^LSS, leaf scorching score; CC, chlorophyll content measured by SPAD meter; LCaC, leaf calcium content; LPC, leaf potassium content; LSC, leaf sodium content; LCC, leaf chloride content; LNaK; leaf sodium to potassium ratio

Clusters 2 and 3 were designated as moderately tolerant and susceptible, respectively, based on mean LSS scores (3.2 and 3.7, respectively). Germplasm accessions in Cluster 4 displayed the highest degree of susceptibility to salinity stress, characterized by a higher mean LSS (4.2) and elevated mean sodium and chloride content in the leaf (Mean LSC: 34.2 g kg^−1^, Mean LCC: 9.2 g kg^−1^). The susceptibility of this cluster was further supported by the presence of the well-documented salt-sensitive cultivar Jackson (PI548657) (Do et al. 2019). Detailed information on germplasm accession in each cluster is presented in Table S2.

### Marker density, linkage disequilibrium, and population structure

Linkage disequilibrium across 20 chromosomes was studied using a SNP dataset comprising 32,832 SNPs. Basic SNP statistics revealed the highest number of SNPs on chromosome 18 (2408), followed by chromosome 13 (2112 SNPs). In contrast, the lowest number of SNPs was observed on chromosome 11 (1274 SNPs) and chromosome 20 (1281 SNPs). Chromosome 13 exhibited the highest marker density with an average of one SNP every 21.7 kb and chromosome 1 showed the lowest marker density with an average of one SNP per 41.3 kb. The average marker density across all chromosomes was approximately one SNP per 29.0 kb (Table S3).

**Fig. 2:**
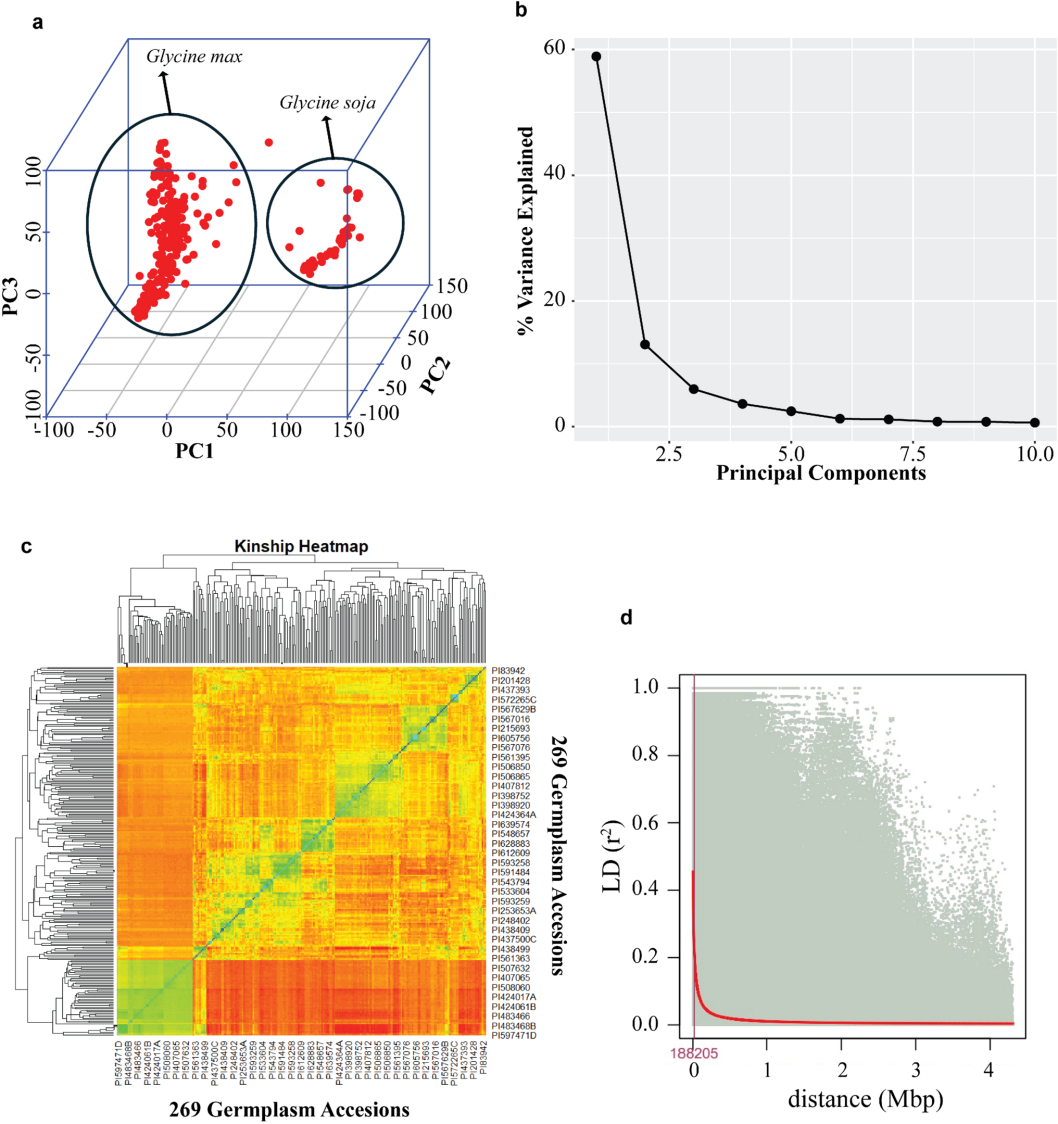
Population structure, PCA, and linkage decay analysis. **a** Three-dimensional PCA plot corresponding to 269 diverse soybean germplasm accessions. **b** Scree plot depicting the variance explained by the top 10 principal components. **c** Kinship heatmap of 269 diverse soybean germplasm accessions. **d** Linkage disequilibrium (LD) decay across all chromosomes. The x-axis and y-axis represent the distance (bp) and LD, respectively. The intersection of red and maroon lines indicates the distance at r^2^ dropped to half its maximum value

To account for potential population substructure, PCA was conducted using SNP markers and identified two distinct sub-populations, corresponding to *Glycine max* and *Glycine soja*. The PC1 and PC2 explained 60.9% and 12.4% of the total variance, respectively (Fig. 2a–b). The cryptic relationship among soybean accessions was examined through a kinship matrix derived from identity-by-state (IBS) calculations for each pair of soybean lines. The heatmap, designed to illustrate this kinship matrix, effectively verified the distinct clustering of *Glycine max* and *Glycine soja* accessions (Fig. 2c). The average half-decay distance, which indicates the distance at which LD drops by half, was approximately 188 kb within the population (Fig. 2d). Additionally, average r^2^ values, a measure of LD, were calculated for each chromosome. These values varied across chromosomes, ranging from 0.143 (chromosome 3) to 0.193 (chromosome 12) (Table S3). The LD decay in our germplasm panel was more pronounced on chromosome 3 compared to chromosome 12 in the soybean genome.

### Genome-wide marker-trait analysis

**Fig. 3:**
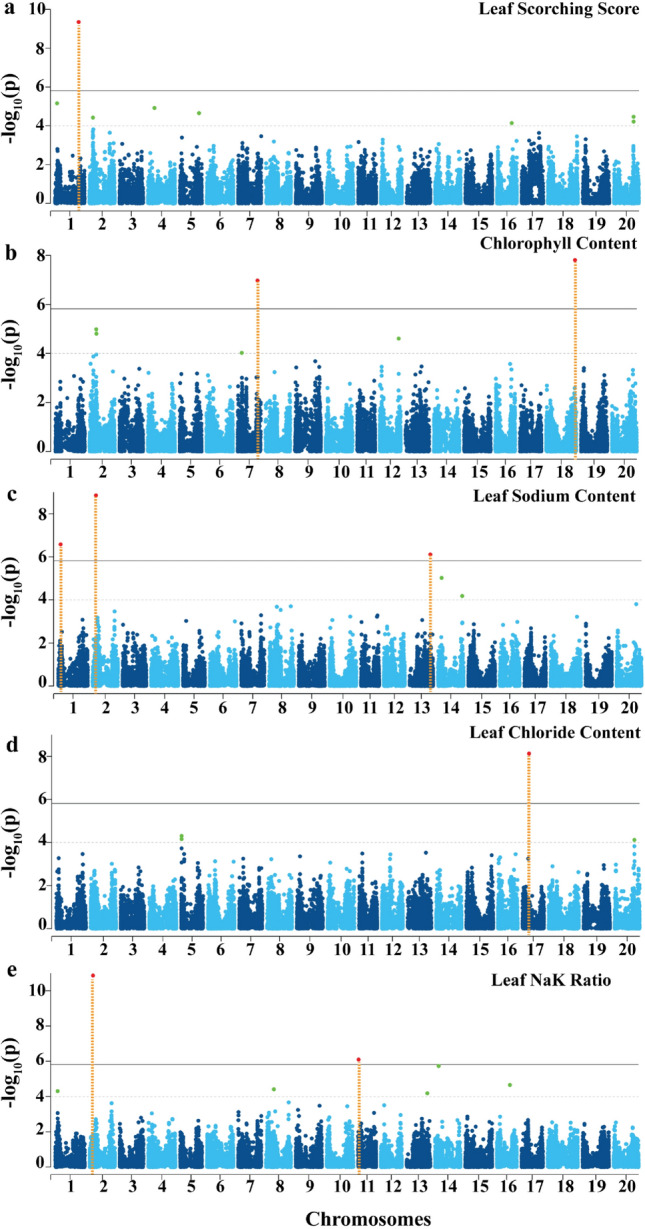
Manhattan plot highlighting significant SNP markers for five different salt-related traits. **a** Leaf Scorching Score (LSS), **b** Chlorophyll Content (CC), **c** Leaf Sodium Content (LSC), **d** Leaf Chloride Content (LCC), and **e** Leaf NaK Ratio (LNaK). The black solid and dotted lines on the Manhattan plot indicate Bonferroni's correction and the manually selected threshold value, respectively

GWAS was performed using the BLINK model to investigate significant MTAs by incorporating kinship (K) and population structure (Q) as covariates. A total of 36 SNPs showed significant trait associations across 15 different chromosomes, with a −log10(P) exceeding 4.0 (Table 3; Fig. 3; Fig. S1). These significant SNPs explained phenotypic variance ranging from 3.6 to 18.8%, with corresponding −log10(P) values varying from 4.0 to 10.9. Within the set of 36 significant MTAs, 11 were identified as highly significant MTAs surpassing Bonferroni's cutoff threshold (Table 4; Fig. 3; Fig. S1).

**Table 3:** SNP markers associated with various morphological and physiological traits under salinity stress [− log(P value) > 4]

Trait^a^	SNP_ID	Chr^b^	Position on the Chr	Allele Present^c^	−log (P value)^d^	MAF^e^	Effect	PVE^f^ (%)	Total PVE (%)
LSS	ss715579060	1	3,404,910	T/C	5.16	0.18	−0.23	8.8	41.5
	ss715579500	1	45,269,288	C/T	9.35	0.22	0.33	9.1	
	ss715583766	2	6,736,648	T/C	4.42	0.11	0.31	12.1	
	ss715587072	4	12,422,768	C/A	4.92	0.09	−0.33	16.8	
	ss715591351	5	36,814,419	T/C	4.65	0.35	−0.25	9.0	
	ss715623903	16	28,102,771	C/T	4.13	0.28	0.21	4.4	
	ss715637881	20	37,773,473	A/G	4.47	0.30	0.24	4.7	
	ss715637883	20	37,786,349	G/A	4.22	0.43	0.19	5.6	
CC	ss715581110	2	12,717,782	A/G	4.98	0.29	1.77	12.8	24.4
	ss715581123	2	12,995,752	A/G	4.81	0.24	1.45	10.5	
	ss715598716	7	7,888,303	C/T	4.03	0.31	0.94	6.1	
	ss715597755	7	38,326,873	A/G	6.97	0.50	−1.01	4.9	
	ss715612654	12	36,308,899	T/C	4.61	0.45	1.06	7.4	
	ss715632259	18	55,901,509	C/T	7.81	0.27	−1.45	8.6	
LCaC	ss715599264	8	11,197,921	G/A	4.40	0.19	0.66	5.5	27.7
	ss715602419	8	46,710,188	T/C	7.24	0.48	−0.73	15.1	
	ss715617622	14	12,652,653	G/A	5.01	0.48	−0.48	7.6	
	ss715622057	15	46,695,426	A/G	6.13	0.41	−0.54	4.5	
LPC	ss715599716	8	15,567,780	T/C	4.69	0.32	−1.54	7.1	14.3
	ss715637552	20	3,413,871	A/G	4.49	0.40	1.81	10.8	
LSC	ss715579377	1	4,140,959	C/T	6.58	0.38	−2.74	13.5	39.2
	ss715583736	2	6,456,965	G/A	8.85	0.13	3.97	18.2	
	ss715616176	13	40,244,469	A/G	6.11	0.22	−2.93	8.8	
	ss715619793	14	6,741,624	T/C	5.02	0.36	2.20	7.6	
	ss715619274	14	46,567,312	C/T	4.18	0.36	2.11	9.1	
LCC	ss715592539	5	595,812	G/A	4.31	0.49	−0.53	6.5	24.0
	ss715592536	5	624,477	A/G	4.15	0.40	−0.46	7.4	
	ss715625956	17	12,795,804	C/T	8.13	0.33	0.73	10.8	
	ss715637886	20	37,805,734	A/C	4.12	0.44	−0.43	4.3	
LNaK	ss715579377	1	4,140,959	C/T	4.31	0.38	−0.07	10.6	52.0
	ss715583736	2	6,456,965	G/A	10.86	0.13	0.13	18.8	
	ss715599627	8	14,784,692	T/G	4.41	0.47	0.05	3.6	
	ss715611231	11	847,393	A/G	6.10	0.26	−0.12	17.7	
	ss715616176	13	40,244,469	A/G	4.18	0.22	−0.06	9.2	
	ss715619801	14	6,769,526	C/T	5.73	0.38	−0.06	6.5	
	ss715623798	16	24,962,131	G/A	4.65	0.41	0.06	7.8	

^a^LSS, leaf scorching score; CC, chlorophyll content; LCaC, leaf calcium content; LPC, leaf potassium content; LSC, leaf sodium content; LCC, leaf chloride content; LNaK; leaf sodium to potassium ratio

^b^Chr, Chromosome; ^c^Allele present; reference or alternate allele at identified SNP i.e., C/T C = reference allele, T = alternate allele; ^d^-log (P.value), negative log of p value; ^e^MAF, minor allele frequency; ^f^PVE, phenotypic variation explained by each marker

**Table 4:** Highly significant SNP markers associated with various morphological and physiological traits under salinity stress [− log (P value) > Bonferroni’s Correction]

Trait^a^	SNP_ID	Chr^b^	Position	Allele Present^c^	−log (P value)^d^	MAF^e^	PVE^f^ (%)
LSS	ss715579500	1	45,269,288	C/T	9.35	0.22	9.1
CC	ss715597755	7	38,326,873	A/G	6.97	0.50	4.9
CC	ss715632259	18	55,901,509	C/T	7.81	0.27	8.6
LCaC	ss715602419	8	46,710,188	T/C	7.24	0.48	15.1
LCaC	ss715622057	15	46,695,426	A/G	6.13	0.41	4.8
LSC	ss715579377	1	4,140,959	C/T	6.58	0.38	13.5
LSC	ss715583736	2	6,456,965	G/A	8.85	0.13	18.2
LSC	ss715616176	13	40,244,469	A/G	6.11	0.22	8.8
LCC	ss715625956	17	12,795,804	C/T	8.13	0.33	10.8
LNaK	ss715583736	2	6,456,965	G/A	10.86	0.13	18.8
LNaK	ss715611231	11	847,393	A/G	6.10	0.26	17.7

^a^LSS, leaf scorching score; CC, chlorophyll content; LCaC, leaf calcium content; LPC, leaf potassium content; –LSC, leaf sodium content; LCC, leaf chloride content; LNaK; leaf sodium to potassium ratio

^b^Chr, Chromosome; ^c^Allele present; reference or alternate allele at identified SNP i.e., C/T C = reference allele, T = alternate allele; ^d^ −log (P.value), negative log of p value; ^e^MAF, minor allele frequency; ^f^PVE, phenotypic variation explained by each marker

**Fig. 4:**
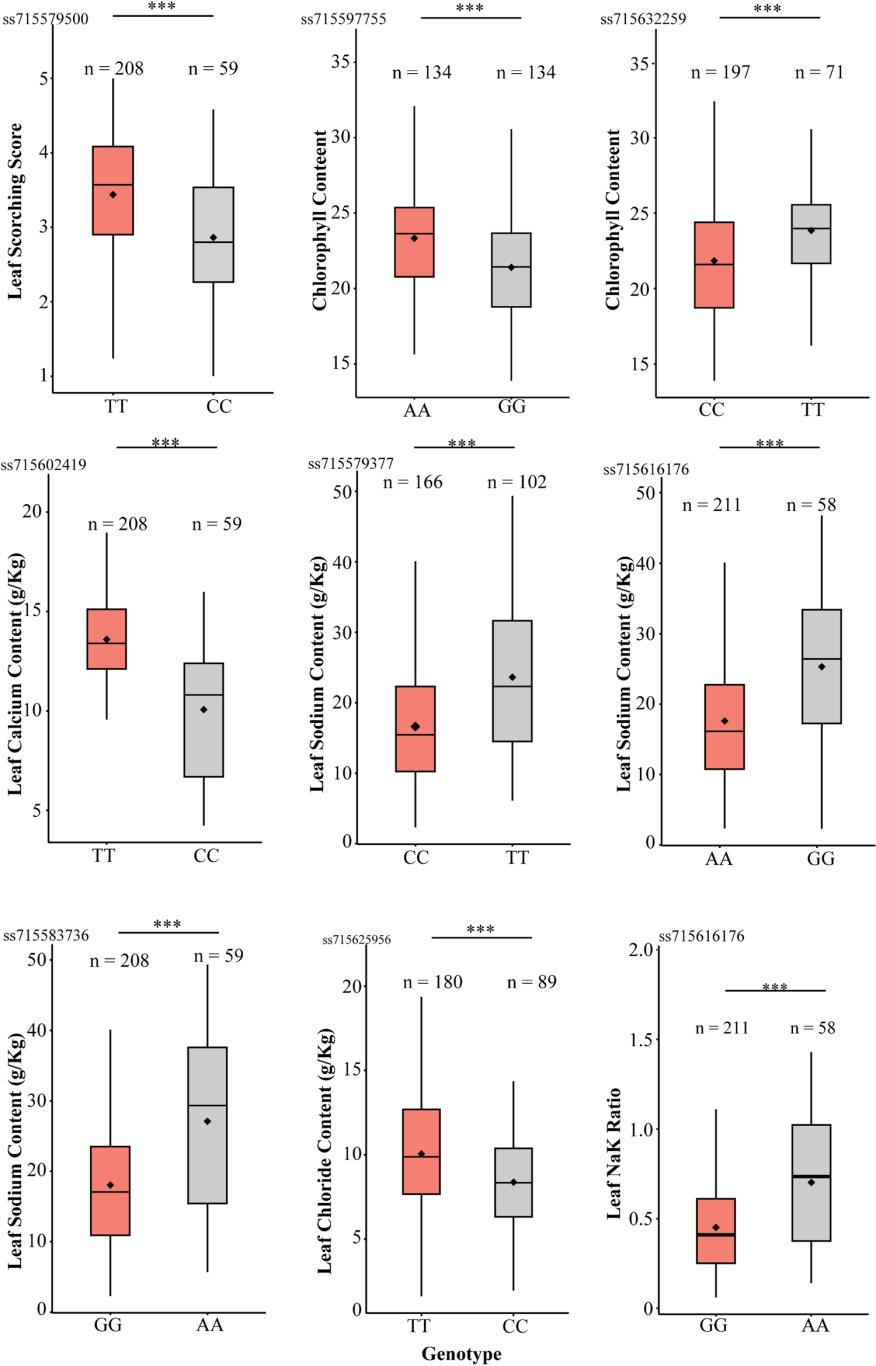
Phenotypic distribution of germplasm accessions across the alleles of highly significant SNP markers such as ss715579500 (LSS), ss715597755 (CC), ss715632259 (CC), ss715602419 (LCaC), ss715579377 (LSC), ss715516176 (LSC), ss715583736 (LSC), ss715625956 (LCC), and ss715616176 (LNaK)

In this study, we identified a significant MTA (ss715579500) on chromosome 1 for LSS, with a −log10(P) value of 9.35. Two other highly significant MTAs, ss715597755 and ss715632259, linked to CC, were identified on chromosome 7 (− log10(P) = 6.97) and chromosome 18 (− log10(P) = 7.81), respectively. Chromosome 2 harbored a significant MTA (ss715583736) associated with both the LNaK ratio and LSC, explaining 18.8% and 18.2% of the phenotypic variation, respectively. A single highly significant MTA (ss715625956, log10(P) = 8.13) on chromosome17 explained 10.8% of the phenotypic variation for LCC. Other highly significant MTAs were identified on chromosome 1 (ss715579377), chromosome 8 (ss715602419), and chromosome 11 (ss715611231) for LSC, LCC, and LNaK ratio, respectively. The phenotypic distribution of alleles across these highly significant SNP markers is illustrated in Fig. 4.

The proportion of phenotypic variation explained by significant markers varied considerably across traits. There were 8 and 9 significant MTAs collectively explaining 52.0% and 41.5% of the variation for LNaK and LSS, respectively. In contrast, MTAs for LPC and LCC explained only 14.3% and 24% of the variation, respectively.

### Haplotype analysis

**Fig. 5:**
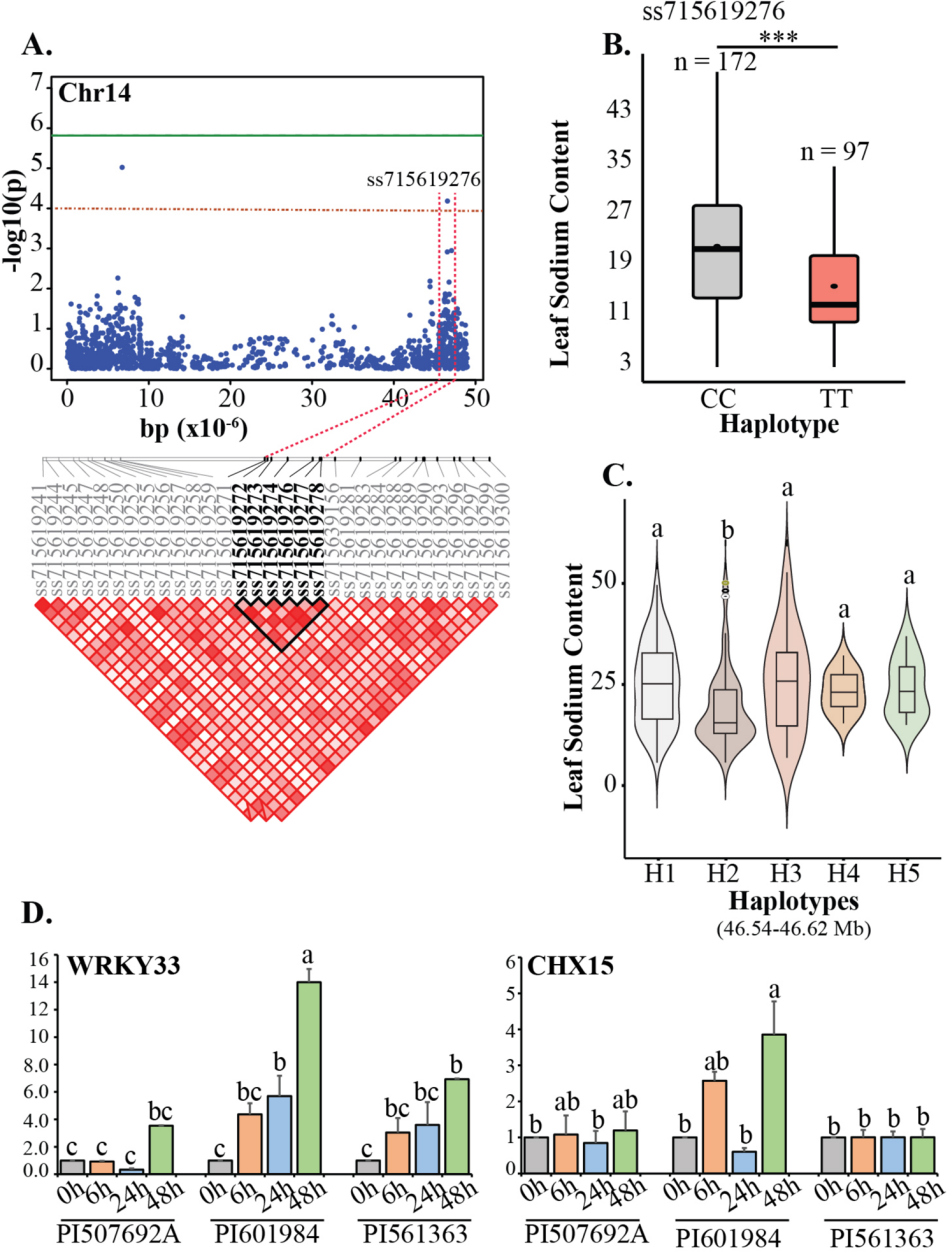
Candidate gene and haplotype analysis across the significant marker (ss715619276) identified on chromosome 14. **a** Local Manhattan plot (top) and LD heatmap (bottom) surrounding the peak on chromosome 14 for leaf sodium content (LSC). Dashed red lines indicate the candidate region for the peak. The thick black line on the LD heatmap represents the high LD region surrounding the significant MTA. **b** Phenotypic distribution of germplasm accessions across the alleles of ss715619276. **c** Haplotypes present within the high LD region of ss715619276. **d** Expression pattern of candidate genes, *WRKY33* and *CHX15*, in two salt-tolerant accessions (PI 507692A and PI 561363) and salt-susceptible accession (PI 601984). Both genes are in the high LD region of ss715619276. Relative change in gene expression levels was determined using the 2^–∆∆CT^ method. Means with a similar letter are not significantly different, according to Tukey test (*p* < 0.05). Error bars represent mean ± SD. *** represents significance at 0.001 probability level

The haplotype analysis was conducted to identify specific combinations of alleles at multiple loci that are inherited together and to determine their association with salt tolerance in soybean. We pinpointed a significant marker associated with LSC near the terminal region of chromosome 14. The marker ss715619274 explained 9.1% of the phenotypic variation for LSC (Fig. 5a). Analysis of the phenotypic distribution across each allele for the significant MTA revealed that germplasm accessions with the minor allele (TT) exhibited markedly less Na^+^ accumulation, averaging 15.2 g kg^−1^ (Fig. 5b). Conversely, accessions with the major allele (CC) demonstrated significantly higher Na^+^ accumulation, averaging 21.5 g kg^−1^ (Fig. 5b). Using high pairwise LD correlations, we determined a candidate region from 46.51 to 46.62 Mb (Fig. 5a; Fig. S2a). The significant MTA, along with the other SNPs within this location, underwent haplotypic analysis. We categorized all the polymorphisms in the candidate region into five distinct categories (Fig. 5c). Haplogroup1 (H1) contained 91 germplasm accessions with the TCTCTC sequence across the SNPs identified in the haploblock. Similarly, haplogroup2 (H2) contained 75 germplasm accessions with the TTTTCC sequence across the SNPs present in the haploblock. All other haplogroups were relatively smaller in size, with germplasm accessions varying from 12 (H5) to 26 (H3). The germplasm accessions present within H2 showed a significant reduction in sodium uptake compared to germplasm accessions present in other haploblocks. Within this haploblock region, two different causal genes, *Glyma.14G200200* (*GmWRKY33*), and *Glyma.14G200100* (*GmCHX15*), were identified, both having roles during salinity stress. Both genes showed significant upregulation in susceptible accessions at 48 h following salt stress exposure in contrast to the observed expression in tolerant accessions (Fig. 5d).

**Fig. 6:**
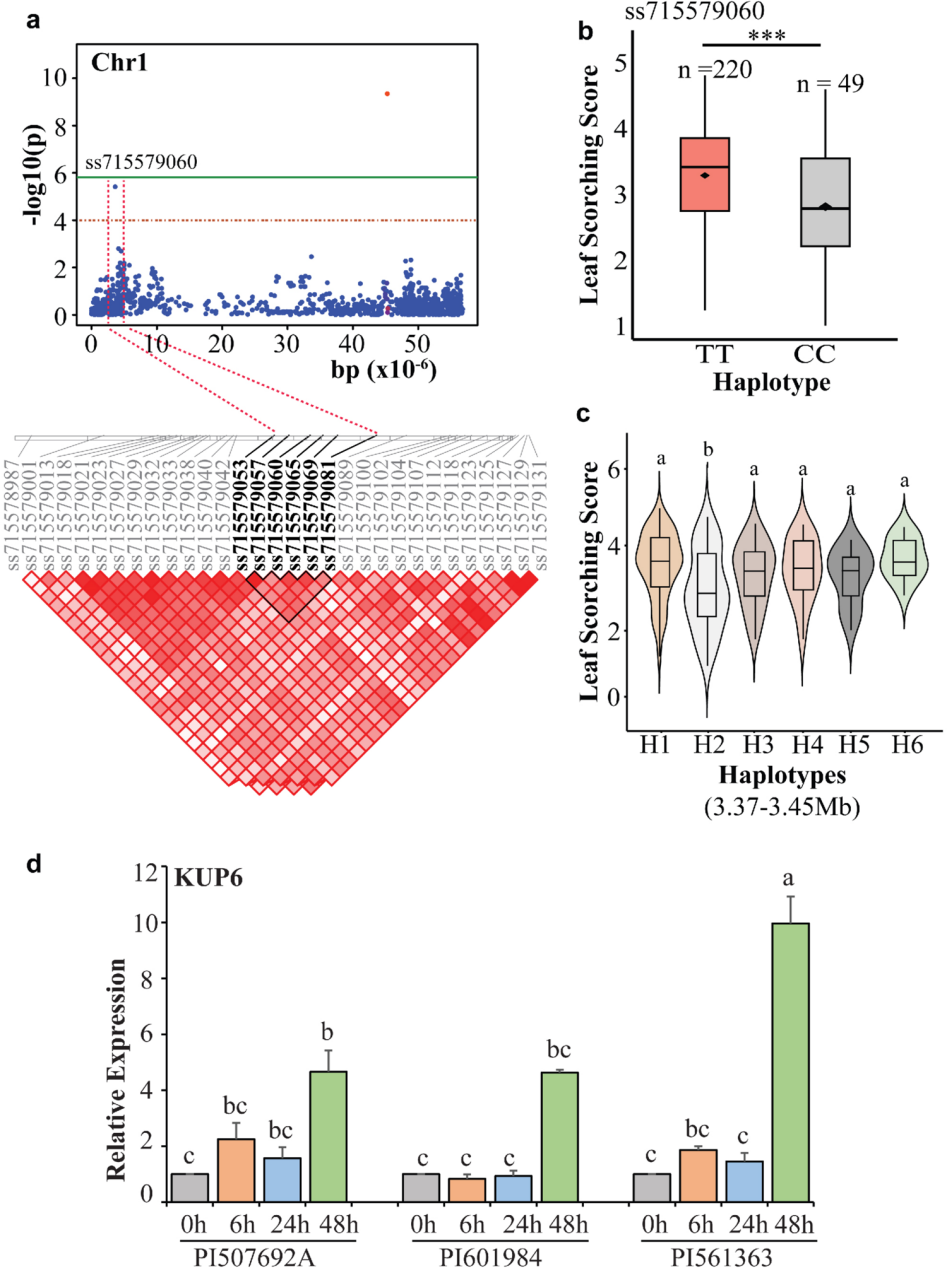
Candidate gene and haplotype analysis across the significant marker (ss715579060) identified on chromosome 1. **a** Local Manhattan plot (top) and LD heatmap (bottom) surrounding the peak on chromosome 1 for leaf scorching score (LSC). Dashed red lines indicate the candidate region for the peak. The thick black line on the LD heatmap represents the high LD region surrounding the significant MTA. **b** Phenotypic distribution of germplasm accessions across the alleles of ss715579060. **c** Haplotypes present within the high LD region of ss715579060. **d** Expression pattern of candidate gene *KUP6* (potassium transporter 6) in the high LD region of ss715579060. Relative change in gene expression levels was determined using the 2^–∆∆CT^ method. Means with a similar letter are not significantly different according to Tukey test (*p* < 0.05). Error bars represent mean ± SD. *** represents significance at 0.001 probability level

Similarly, a peak identified on chromosome 1 explained 8.8% phenotypic variation for LSS (Fig. 6a). The observed phenotypic distribution for each allele in the significant MTA (ss715579060) indicated that germplasm accessions with the minor allele (CC) exhibited a lower average LSS (2.9) (Fig. 6b). Moreover, accessions carrying the major allele (TT) recorded a higher average LSS (3.5). We identified a candidate region ranging from 3.37 to 3.45 Mb using strong pairwise LD correlations, and 6 haplogroups were identified in this region (Fig. 6c; Fig. S2b). Haplogroup2 (H2; n = 41) with a sequence of ACCAGC across different SNP markers showed a significant reduction in LSS. Analysis of candidate genes revealed the presence of the potassium transporter 6 (*GmKUP6*) gene near this region. This gene showed significant upregulation in one of the tolerant accessions (PI 561363) compared to other tolerant (PI 507692A) and susceptible germplasm accession (PI 601984) (Fig. 6d). PI 561363 belonged to H2, whereas PI 601984 and PI 507692A were associated with different haplogroups. Therefore, this difference in distribution might be contributing to varying gene expression across the three germplasm accessions.

### Gene ontology and qRT-PCR analysis

**Fig. 7:**
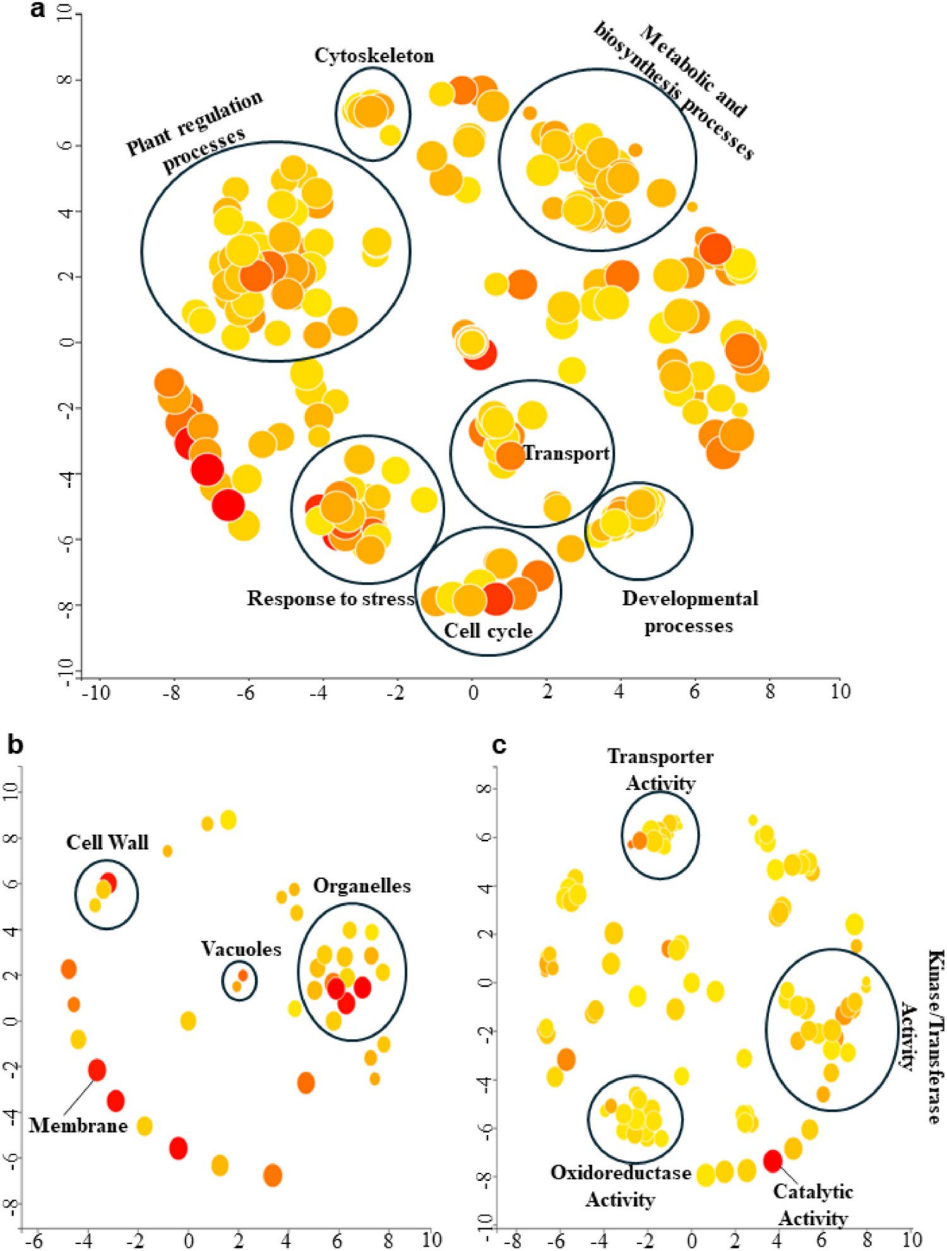
Gene Ontology (GO) enrichment analysis depicting the involvement of candidate genes in three major plant processes. **a** Biological Processes (BP). **b** Cellular Component (CC). **c** Molecular Function (MF)

To uncover the biological functions underlying the identified MTAs, we performed GO analysis for 584 genes within a 100 kb region flanking each significant marker (Table S4). This analysis revealed a broad spectrum of biological processes (BP), cellular components (CC), and molecular functions (MF) terms associated with the MTAs (Fig. 7). BP terms such as plant regulation, cytoskeleton organization, transport mechanisms, developmental processes, metabolic and biosynthesis processes, cell cycle regulation, and responses to stress were significantly enriched. CC terms were enriched with cell wall dynamics, vacuole-related processes, organelle functions, and membrane activities, while MF terms were enriched with categories such as transporter activities, kinase/transferase functions, oxidoreductase activities, and catalytic activities (Table S5).

GO analysis also revealed genes enriched in processes related to abiotic stress, particularly response to salinity, hyperosmotic salinity response, and response to osmotic stress. This analysis also confirmed the presence of these processes under the subcategory response to stress (Fig. 7). Further analysis revealed enrichment of genes such as *GmCA1* (*Glyma.05G007100*), *GmCBL10* (*Glyma.08G194000*), *GmPLDEPSILON* (*Glyma.08G194100*), *GmWRKY33* (*Glyma.14G200200*), *GmPIP2B* (*Glyma.02G073600*), *GmGS2* (*Glyma.02G127500*), *GmCHX15* (*Glyma.14G200100*), *GmKUP6* (*Glyma.01G031800*), *GmRCI3* (*Glyma.13G307000*), *GmNRT1*.5 (*Glyma.17G153300*), and *GmLTP4* (*Glyma.01G132500*), *GmRAP2.11* (*Glyma.02G072800*), and *GmCRK29* (*Glyma.20G138400*), highlighting their potential role in influencing the observed phenotypic variation due to their involvement in specific stress response pathways.

**Fig. 8:**
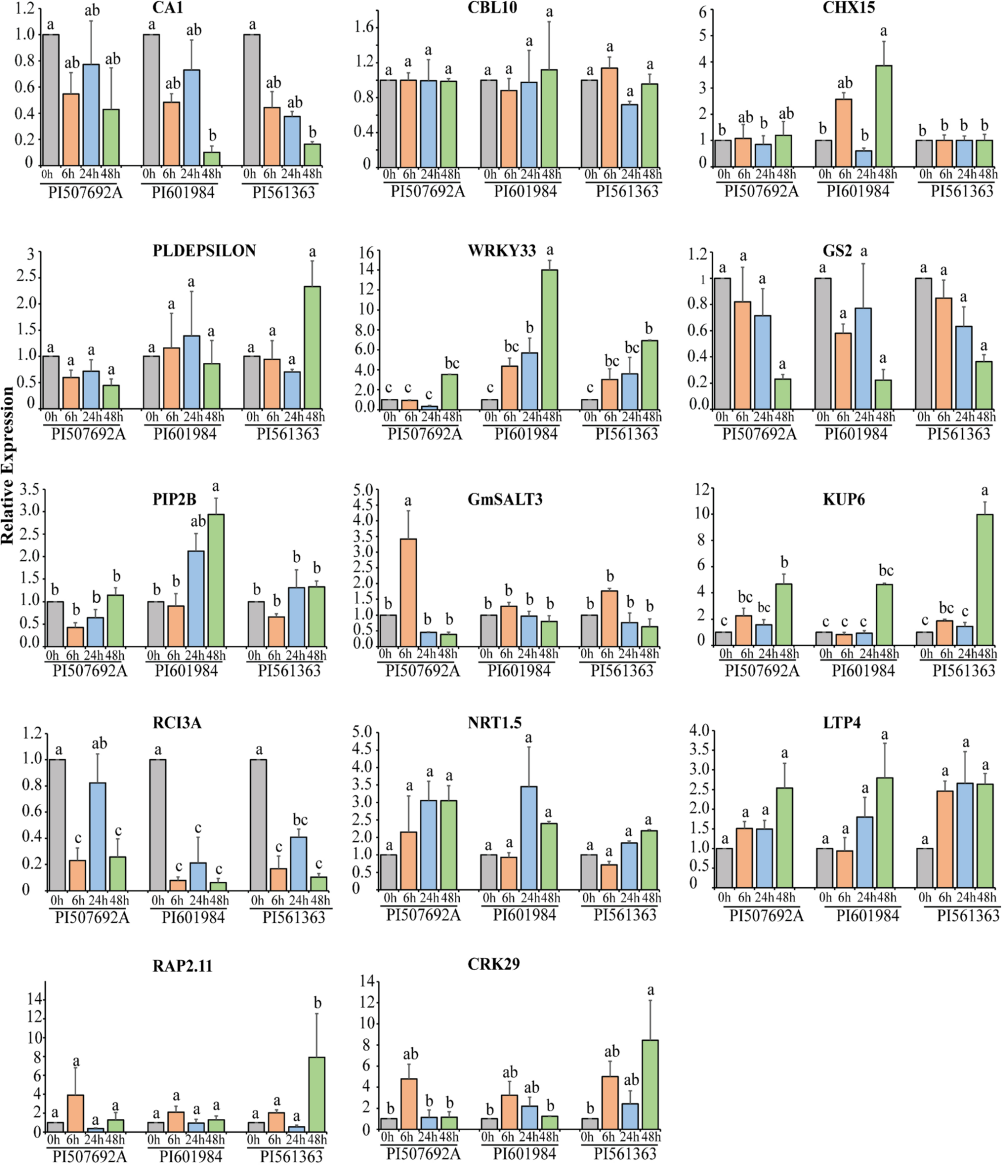
Expression analysis of selected candidate genes in three different germplasm accessions, PI 507692A (Tolerant), PI 601984 (Susceptible), and PI 561363 (Tolerant), at 0 h, 6 h, 24 h, and 48 h after imposition of salt stress. The y-axis represents the relative change in mRNA expression level, while the x-axis depicts the different time points and genotypes

To validate the roles of candidate genes in response to salinity stress across three genotypes (PI 507692A, PI 601984, PI 561363) at 0 h, 6 h, 24 h, and 48 h, qRT-PCR was performed (Fig. 8; Table S6). The stress-responsive gene *GmWRKY33* showed significant upregulation only in the susceptible genotype at 48 h, potentially linking it to susceptibility. Conversely, the potassium transporter *GmKUP6* displayed upregulation across all genotypes under salt stress, but the tolerant genotype (PI 561363) exhibited a remarkably higher expression (ten-fold). Additionally, genes like *GmPIP2B* and *GmCHX15* displayed increased expression in the susceptible genotype at 48 h. Still, their expression remained unchanged in tolerant accessions at all time points, highlighting stress response differences between genotypes.

### Genomic selection

**Fig. 9:**
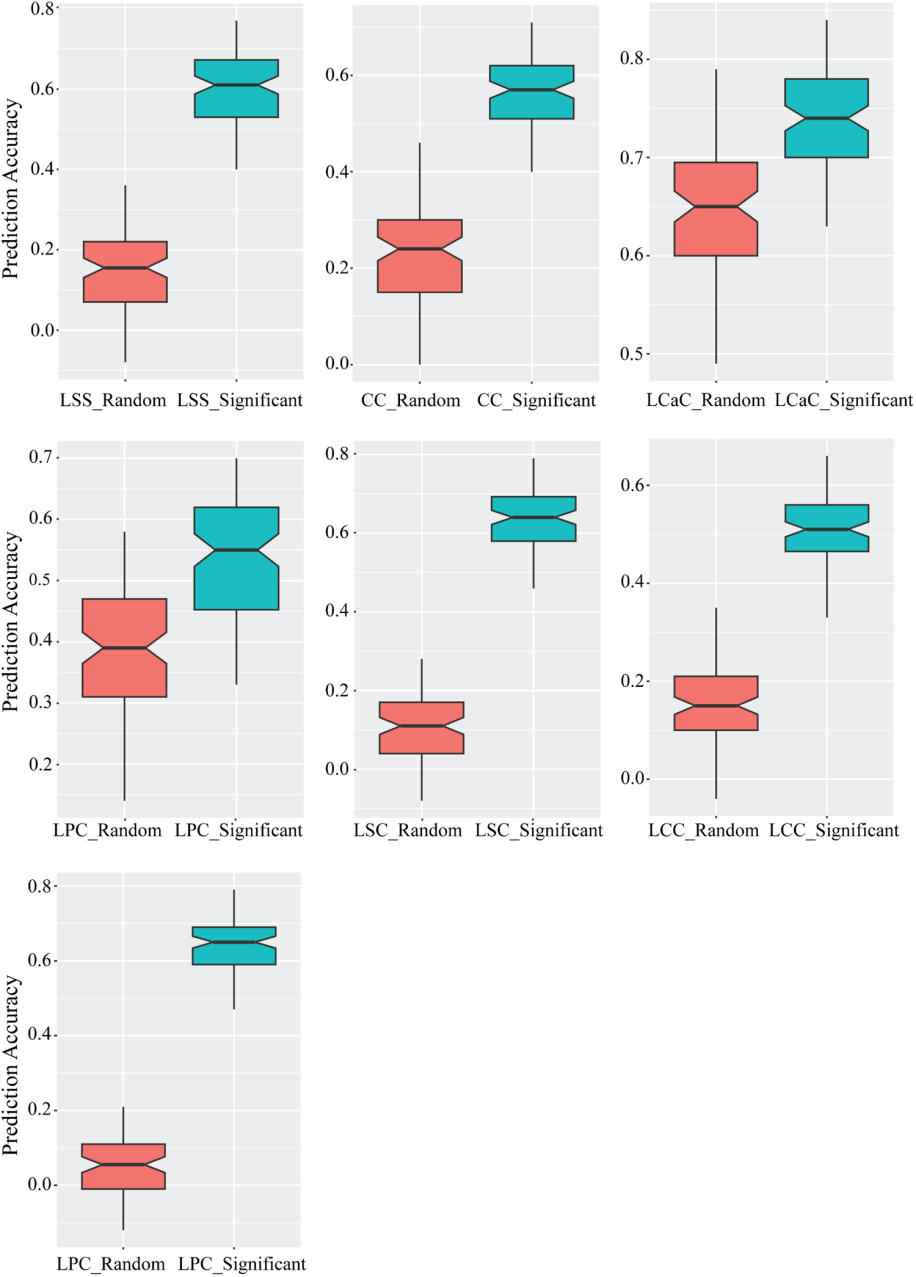
Genomic prediction using GBLUP method. The y-axis depicts prediction accuracy, while x-axis represents the random markers and markers significantly associated with various morpho-physiological traits under salt stress

In our diverse soybean panel, we identified 36 markers linked to various salinity-related traits. Among them, three markers (ss715579377, ss715583736, ss715616176) overlapped for two distinct traits (LSC and LNaK), while one marker exhibited significant LD (r^2^ > 0.8) with another. Consequently, these markers were excluded from further analysis. This left us with 32 significant markers and 32 randomly selected markers distributed across different chromosomes for genome-based prediction. Using these significant markers, GBLUP yielded varying prediction accuracies ranging from 0.5 (for LCC) to 0.74 (for LCaC), as illustrated in Table 5 and Fig. 9. Conversely, the prediction accuracy was notably lower when randomly selected markers were employed: LSS (0.15), CC (0.23), LSC (0.10), LCC (0.15), and LNaK (0.05).

**Table 5:** Prediction accuracy and marker-based narrow sense heritability for different salt-related traits

Traits^a^	Markers	h^2^b	PA^c^	BIC^d^
LSS	Significant Markers	0.40 ± 0.03	0.60 ± 0.08	144.60 ± 8.08
	Random Markers	0.04 ± 0.02	0.15 ± 0.10	216.80 ± 1.16
CC	Significant Markers	0.37 ± 0.03	0.56 ± 0.08	157.01 ± 6.81
	Random Markers	0.07 ± 0.02	0.23 ± 0.11	211.44 ± 2.73
LCaC	Significant Markers	0.46 ± 0.02	0.74 ± 0.05	74.83 ± 9.51
	Random Markers	0.33 ± 0.03	0.63 ± 0.10	127.23 ± 10.94
LPC	Significant Markers	0.28 ± 0.03	0.53 ± 0.11	163.97 ± 8.14
	Random Markers	0.13 ± 0.02	0.37 ± 0.12	193.88 ± 6.01
LSC	Significant Markers	0.44 ± 0.03	0.63 ± 0.08	136.89 ± 9.18
	Random Markers	0.03 ± 0.02	0.10 ± 0.10	218.38 ± 1.17
LCC	Significant Markers	0.28 ± 0.02	0.50 ± 0.08	172.60 ± 5.93
	Random Markers	0.06 ± 0.02	0.15 ± 0.10	216.87 ± 1.63
LNaK	Significant Markers	0.46 ± 0.03	0.63 ± 0.09	136.66 ± 9.22
	Random Markers	0.03 ± 0.02	0.05 ± 0.08	218.94 ± 0.80

^a^LSS, leaf scorching score; CC, chlorophyll content; LCaC, leaf calcium content; LPC, leaf potassium content; LSC, leaf sodium content; LCC, leaf chloride content; LNaK; leaf sodium to potassium ratio

^b^h^2^, marker-based narrow sense heritability; ^c^PA, Prediction Accuracy; ^d^BIC, Bayesian information criteria

The marker-based narrow-sense heritability stood at approximately 40% for all traits except LPC, which was slightly lower at 28%. However, there was a significant decrease in narrow-sense heritability observed with random markers across most traits, except for LCaC.

## Discussion

Salinity stress presents a significant challenge to improving crop production in soybean-growing regions worldwide. However, developing salt tolerance in soybean through breeding is a formidable task due to the polygenic nature of this complex trait. The challenge is further compounded by the various mechanisms employed by plants to adapt to saline environments. Information on genomic regions associated with salt tolerance during the seedling stage in soybean has been limited. Thus, we established a diversity panel comprising 269 wild and cultivated soybean germplasm accessions for a GWAS aimed at identifying candidate genes that confer salinity tolerance at the seedling stage.

Assessing salt tolerance in soybean relied on analyzing sodium and chloride ion uptake and morpho-physiological responses to saline stress. Salt-tolerant soybean genotypes typically show lower chloride (Cl^−^) levels in their leaves, leading to increased photosynthate production. Our observation of a significant positive correlation between LSS and LCC and a significant negative correlation between CC and LCC supports this notion. Sodium uptake is equally detrimental to soybean under salt stress, leading to decreased absorption of other essential nutrients required for various biosynthetic processes. This was further confirmed by the significant positive association between LSC and LSS (Table 1). Furthermore, the excessive amount of sodium in the shoots indirectly affects the upward movement of potassium and calcium in the plant, as evidenced by the significant negative correlation of LSC with LCaC and LPC (Table 1).

Genetic analysis of the soybean germplasm accessions revealed two major clusters, as characterized by PCA (Fig. 2a–b). Subsequent kinship analysis consistently confirmed the distinct clustering of the soybean accessions into two groups that corresponded to *Glycine max* and *Glycine soja* (Fig. 2c). Both PC1 and PC2 accounted for 73 percent of the total variation and were employed as covariates in the association mapping analysis to reduce the occurrence of false positives. The effectiveness of the association analysis is influenced by the extent of LD; half LD decay was estimated to be approximately 188 kb which is considerably higher compared to other plant species such as rice (109 kb) and maize (~ 1–10 kb) (Lu et al. 2015; Yan et al. 2009) (Fig. 2d). The cleistogamous nature of soybean may contribute to reduced genomic variation.

**Fig. 10:**
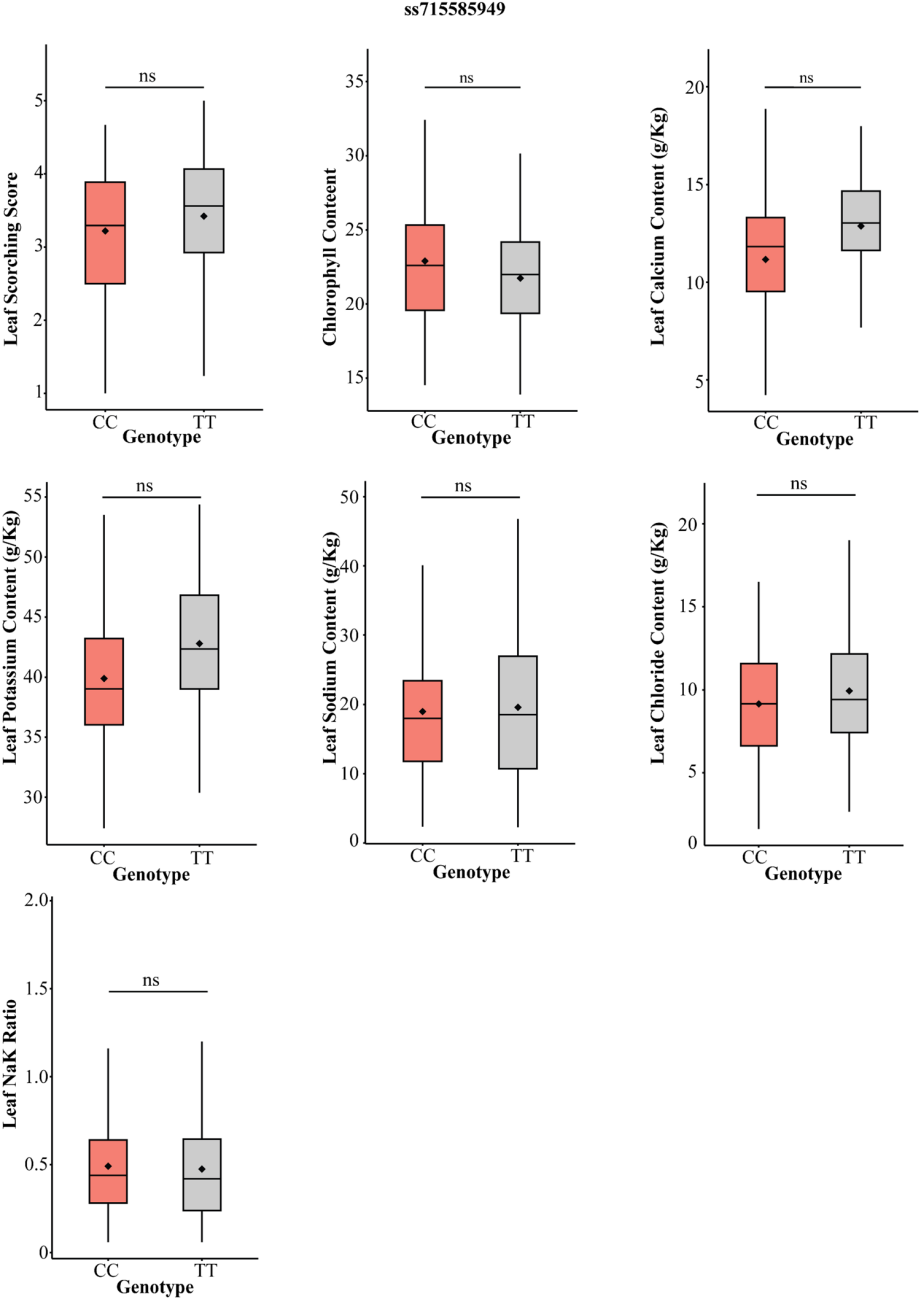
Phenotypic distribution of germplasm accessions across the alleles of ss715585949 for different salt-related traits (marker located adjacent to *GmSALT3* gene on chromosome 3)

Saline stress induces osmotic stress, ionic imbalance, and nutritional deficiencies in plants. Previous studies have shown that the upregulation of stress-responsive transporters and antioxidant production genes during stress conditions can improve plant tolerance to various abiotic stresses. Despite multiple approaches to identifying genes associated with salinity tolerance in soybean, only a limited number of genes have been recognized as key regulators of the soybean's response to salt stress. Multiple studies have consistently reported a significant gene for salt tolerance, *Glyma.03g32900* (*GmSALT3*/*GmCHX1*), which encodes a sodium/hydrogen (Na^+^/H^+^) exchanger family protein (Guan et al. 2014; Qi et al. 2014; Zeng et al. 2017; Do et al. 2019). However, our GWAS analysis did not identify this locus, suggesting the involvement of other genes related to salt tolerance in our germplasm panel. To validate this result, we performed hypothesis testing to assess differences between the alleles of the flanking markers associated with *Glyma.03g32900*. The results revealed no significant difference in crucial salinity traits between the alleles across both flanking markers (Fig. 10; Fig. S3; Table S7). The absence of this gene on chromosome 3 associated with salt tolerance in this study might be due to exclusion of the genotypes used in earlier GWAS and QTL mapping studies, where the significant MTA was identified on chromosome 3. By excluding these genotypes, the germplasm pool used in this study most likely lacked the crucial variants associated with the *GmSALT3* gene. Eleven highly significant SNPs associated with salinity tolerance suggest the involvement of distinct loci at these genomic positions (Table 3; Fig. 3; Fig. S1). The LSS serves as a crucial criterion for evaluating salt tolerance during the seedling stage. Association mapping identified a significant MTA (ss715579500) for LSS on chromosome 1 with −log_10_ (P) value of 9.35. Candidate gene analysis revealed the presence of the lipid transfer protein 4 (*LTP4*) gene, which exhibited increased expression under salt conditions in *Arabidopsis* (Julke and Ludwig-Muller 2015). In *Nicotiana tabacum*, the overexpression of *NtLTP4* also correlated with reduced Na^+^ levels through the upregulation of Na^+^/H^+^ exchangers (*NHX1*) and high-affinity K^+^ transporter1 (*HKT1*) (Xu et al. 2018). We observed the upregulation of *GmLTP4* in both tolerant and susceptible genotypes 48 h after exposure to salt stress (Fig. 8).

In the chromosomal region near another marker (ss715579060) on chromosome 1, a potassium ion transporter (*GmKUP6*) was present. The KUP family, known for its role in potassium (K^+^) uptake, plays a vital role in various physiological processes. Previous studies have demonstrated high-affinity K^+^ uptake and up-regulation in response to low K^+^ conditions for the transporters such as *HvHAK1* in barley, *AtHAK5* in *Arabidopsis*, and *OsHAK1/OsHAK5* in rice (Santa-María et al. 1997; Gierth et al. 2005; Yang et al. 2014). *GmKUP6* displayed a tenfold increase in expression 48 h after salt exposure in the tolerant genotype (PI561363), whereas only a fourfold increase was observed in the susceptible genotype (PI 601984), suggesting the involvement of this gene in maintaining ionic homeostasis.

The marker ss715597755, associated with CC, was identified 300 Kb upstream of the region identified by Zeng et al. (2017). This region encompassed a vacuole-import-associated protein gene (*Glyma.07G212300*) and a chloroplast organization gene, *CYO1,* in *Arabidopsis* (*Glyma.07G21210*). Additionally, we identified ss715632259 for CC on chromosome 18, located close to the MTA identified by Do et al. (2019). Notably, this candidate region encompassed genes (*Glyma.18G276500*, *Glyma.18G278100*) related to photosynthetic activity in *Arabidopsis.*

This study discovered significant MTAs for LCaC on chromosomes 8 and 15. The region on chromosome 15 contained a C2 domain gene *GmC2-148* (*Glyma.15G245600)* which was significantly upregulated (log_2_FoldChange = 4.0) under saline conditions in a previous study (Sun et al. 2021). Additionally, *GmC2-148* transgenic soybean plants demonstrated increased proline content, reduced levels of H_2_O_2_, O^2−^, and malondialdehyde (MDA), along with increased expression of stress-responsive genes (Sun et al. 2021). The MTA linked to LPC content on chromosome 8 harbored a gene, *Glyma.08G194000,* analogous to SOS3-Like Calcium Binding Protein8 (*SCaBP8*)/Calcineurin B-Like10) (*CBL10*) in *Arabidopsis* (Kim et al. 2007). This gene is reported to interact with *SOS2* and various salt-responsive genes including an H^+^/Ca2^+^ antiporter, a tonoplast H^+^ ATPase, and a tonoplast Na^+^/H^+^ antiporter (Cheng et al. 2004; Qiu et al. 2004; Batelli et al. 2007). However, there was no significant variation in the expression levels of *Glyma.08G194000* among different genotypes and treatments in the present study (Fig. 8).

Association mapping analysis identified one common MTA on chromosome 2 for LSC and LNaK. In a 100 kb region surrounding this MTA, there were two candidate genes, *Glyma.02G073600* and *Glyma.02G073700,* analogous to the *OsPIP2* gene in *Oryza sativa*. *PIP2* is known to facilitate water transport, particularly under drought conditions (Bai et al. 2021). Gene expression analysis revealed that the *GmPIP2* expression increased in a salt-sensitive genotype 24 h and 48 h after salt exposure, whereas there was little or no change in expression levels in salt-tolerant genotypes (Fig. 8). The same region contained another gene homologous to *GmRAP2.11*, which is recognized for its role in controlling the *AtHAK5* gene during low potassium conditions (Kim et al. 2012). In this study, this gene showed increased activity in a saline stress-resistant genotype (PI 561363) after 48 h exposure to saline conditions.

The candidate region for a common SNP linked to LSC and LNaK on chromosome 13 included a gene, *Glyma.13G307000,* analogous to a peroxidase family gene in *Arabidopsis* (*RCI3*, AT1G05260). Overexpression of this gene increased reactive oxygen species (ROS) production and elevated expression of *AtHAK5* (Kim et al. 2010). *GmRCI3* showed significant downregulation in all three genotypes, with the largest decrease observed in the susceptible genotype (PI 601984) under saline conditions at 48 h (Fig. 8).

Another MTA, identified on chromosome 14, included two genes, *Glyma.14G200000* and *Glyma.14G200100*, which showed significant homology with the cation/hydrogen exchanger found in *Arabidopsis*. Adjacent to these genes, another gene *Glyma.14G200200*, was part of the well-known stress-responsive WRKY gene family. In this study, both *Glyma.14G200100* (*GmCHX15)* and *Glyma.14G200200* (*GmWRKY33*) were significantly upregulated in a susceptible germplasm accession (PI 601984) at the 48-h time point (Fig. 8). This observation suggests the potential involvement of these genes in coping with the higher degree of damage resulting from saline stress in the susceptible genotype (PI 601984).

Elevated chloride levels significantly affect soybean plants resulting in ion imbalance, nutrient uptake inhibition, leaf burn, and reduced photosynthesis. In this study, we identified a significant marker-trait association for LCC on chromosome 17. Within the candidate region of this MTA, we discovered a *nitrate transporter 1.5* gene, which showed a significant reduction in expression levels under saline conditions in *Arabidopsis* (Chen et al. 2012). However, our qRT-PCR results revealed no significant change in expression levels of *GmNRT1.5* among the three genotypes at different time points of stress exposure. The marker ss715637886 on chromosome 20 featured a cysteine-rich receptor-like kinase (RLK) protein gene (*GmCRK29*) that exhibited increased expression in the tolerant genotype (PI 561363) 48 h after salt exposure. Conversely, in the susceptible genotype, there was a decrease in the expression of this gene from 6 to 48 h.

Due to the quantitative nature of salinity tolerance, identifying and cloning of genes controlling this trait using fine mapping and map-based cloning has been challenging. The complex genetic architecture of salinity tolerance complicates progress in developing salt-tolerant varieties in the breeding programs. Hence, we assessed the effectiveness of genome-based prediction using a subset of 32 markers significantly associated with salt-responsive traits and 32 randomly chosen markers across different chromosomes. The average prediction accuracy of GBLUP with significant GWAS markers ranged from 0.5 to 0.74 for various traits, demonstrating the benefit of utilizing the linked markers in predicting salinity tolerance compared to randomly selected markers. The decline in marker-based narrow-sense heritability for random markers highlights the importance of using markers associated with target traits to accurately capture the genetic variation relevant to salinity tolerance (Fig. 9). Our results indicate that the GWAS markers hold promise for improving selection efficiency and precision in breeding programs. However, while these findings validate the markers within the same germplasm accessions, further validation in diverse and independent populations is necessary to ensure their broader applicability. By confirming the effectiveness of these markers across different genetic backgrounds, breeders can more reliably develop high-yielding, salt-tolerant soybean varieties, ultimately enhancing productivity in saline-affected regions.

## Conclusion

Identifying salinity-tolerant donors and desirable alleles of the salt-tolerant loci is crucial for improving soybean yield in salt-affected areas. The expansion of soybean acreage necessitates enhancing soybean adaptability to a wide range of environments, particularly those prone to salinity. This study evaluated salinity tolerance in a diverse panel of wild and cultivated soybean germplasm accessions, revealing significant variations in their physiological responses to salinity stress. We identified new soybean germplasm accessions, including PI 561363, PI 507692A, PI 468399A, PI 437109C, and PI 639569, that exhibited elevated salinity tolerance. Most of these genotypes with enhanced performance under salt stress showed a tendency toward low-to-moderate sodium Na^+^ and Cl^−^ accumulation. This study provided valuable insights into the genomic regions associated with salinity tolerance at the seedling stage. The MTAs revealed candidate genes involved in ion transport, antioxidant production, and stress response pathways. Furthermore, the study emphasized the importance of utilizing significant markers identified through GWAS for enhancing selection efficiency in breeding programs. Incorporating these markers can accelerate the development of new cultivars with desired salt-tolerance traits, thereby improving the productivity and sustainability of soybean in salt-affected regions.

## Data availability statement

All relevant data are provided as tables and figures in the paper and in the supplementary materials.

## Supplementary Information

Below is the link to the electronic supplementary material.

Supplementary file1 (PDF 574 KB)

Supplementary file2 (XLSX 35 KB)

Supplementary file3 (XLSX 88 KB)

Supplementary file4 (XLSX 44 KB)
