# Supplementary material for: Deciphering the genetic basis of salinity tolerance in a diverse panel of cultivated and wild soybean accessions by genome-wide association mapping
Source: Theor Appl Genet. 2024 Sep 28;137(10):238. doi: 10.1007/s00122-024-04752-4 (PMC11438739; doi:10.1007/s00122-024-04752-4)
Supplement: Supplementary file 1 — Supplementary file1 (PDF 574 KB) [file 122_2024_4752_MOESM1_ESM.pdf]

Supplementary information

Title: Deciphering the Genetic Basis of Salinity Tolerance in a Diverse Panel of Cultivated and Wild Soybean Accessions by Genome-Wide Association Mapping and Genomic Selection

Journal Name: Theoretical and Applied Genetics

Authors: Rajat Pruthi[1], Chanderkant Chaudhary[1], Sandeep Chapagain[1], Mostafa M.E. Abozaid[3], Prabhat Rana[1], Ravi Kiran Reddy Kondi[1], Roberto Fritsche-Neto[2], Prasanta K. Subudhi[1*]

Affiliations: [1]School of Plant, Environmental, and Soil Sciences, Louisiana State University Agricultural Center, Baton Rouge, LA 70803, USA

[2]Rouse Caffey Rice Research Station, LSU AgCenter, Rayne, USA

[3]Botany and Microbiology Department, Faculty of Science, Al-Azhar University, Cairo,11884, Egypt

[*]Corresponding author E-mail address: psubudhi@agcenter.lsu.edu

The Supplementary information includes 3 figures (Fig 1S to Fig S3) and 7 tables (Table S1 to Table S7)

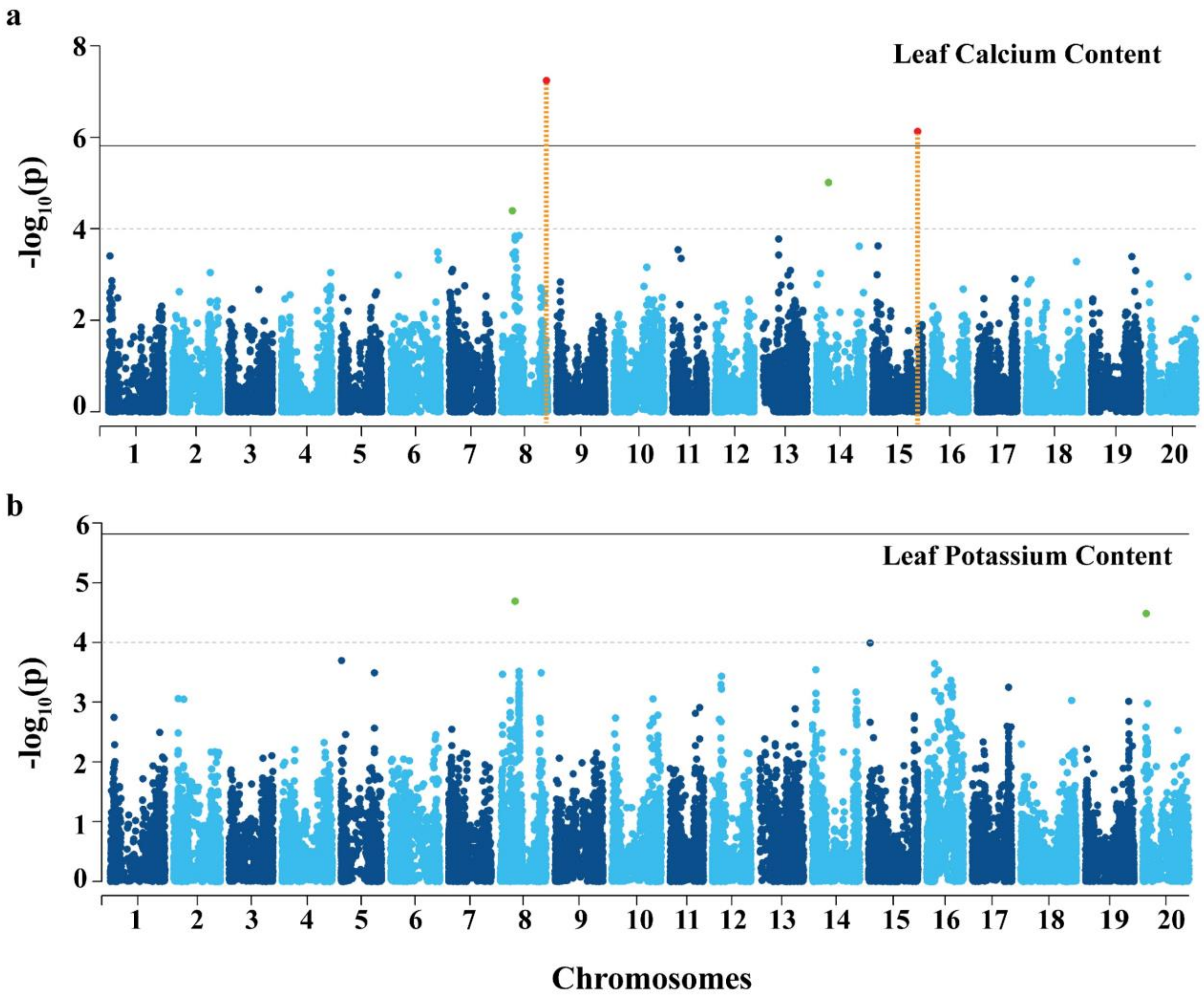

Fig. S1. Manhattan plot highlighting significant SNP markers for two different salt-related traits. (**a**) Leaf Calcium Content (LCaC), (**b**) Leaf Potassium Content (LPC). The black solid and dotted lines on the Manhattan plot indicate Bonferroni's correction and the manually selected threshold value, respectively.

a

	46548120	46548120	46548120	46548120	46548120	46548120	freq
ALLELE	T/G	C/T	T/G	C/T	T/C	C/T	
H001	T	C	T	C	T	C	91
H002	T	T	T	T	C	C	75
H003	G	C	G	C	T	C	26
H004	G	C	T	C	C	C	14
H005	G	C	G	C	C	C	12

b

	3375794	3387651	3404910	3415315	3427220	3458900	freq
ALLELE	A/G	C/A	T/C	G/A	T/G	T/C	
H001	G	A	T	G	T	T	85
H002	A	C	C	A	G	C	41
H003	A	C	T	G	T	C	31
H004	A	C	T	G	T	T	20
H005	A	C	T	G	G	C	15
H006	G	A	T	G	T	C	13

Fig. S2. Sequence and frequency of germplasm accessions in different haplogroups in high LD region of significant MTAs. a) ss715619274 on chromosome 14. b) ss715579060 on chromosome 1.

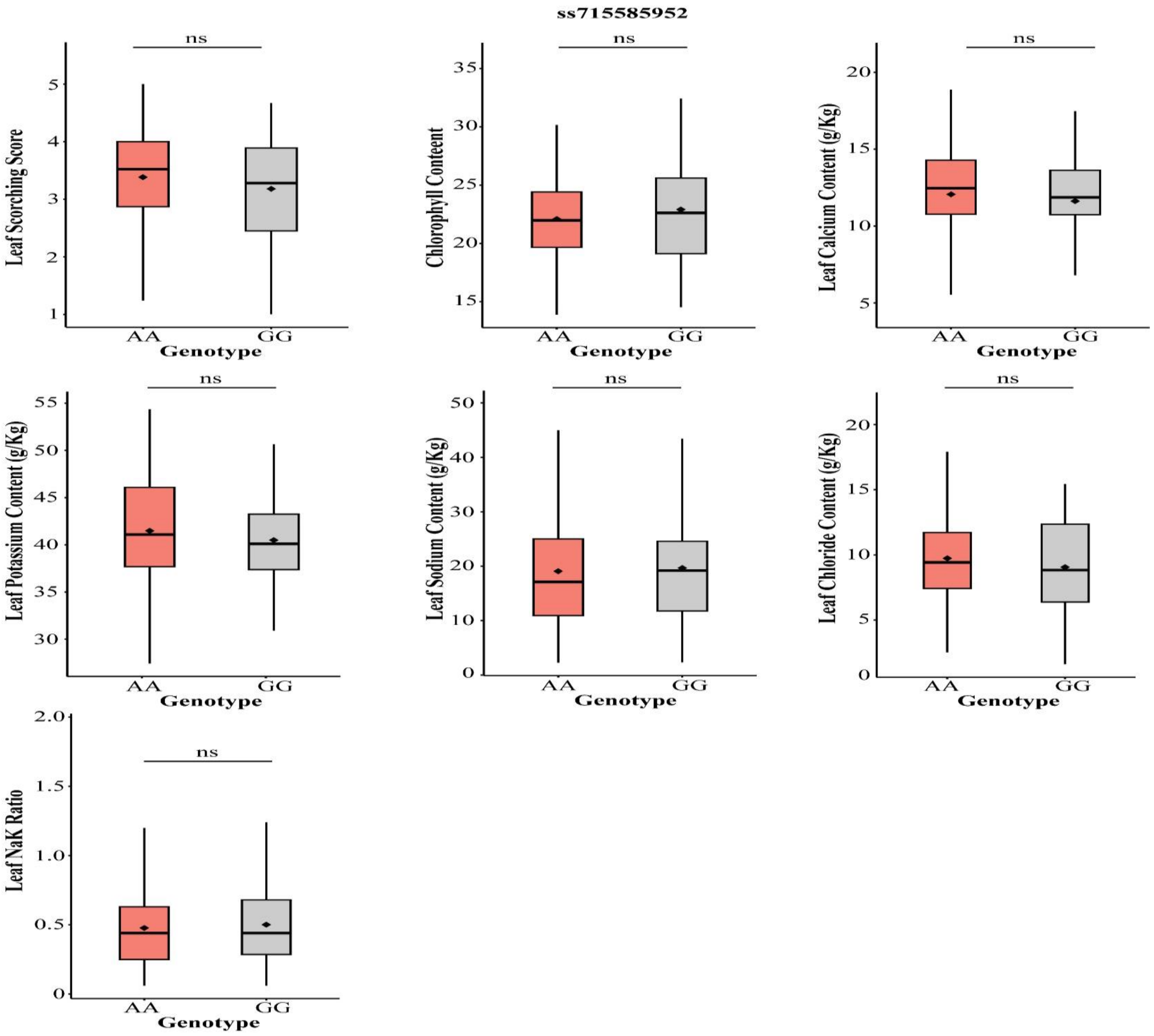

Fig. S3. Phenotypic distribution of germplasm accessions across the alleles of ss715585952 for different salt-related traits (marker located adjacent to *GmSALT3* gene on chromosome 3).

Table S2. Classification of soybean germplasm accessions based on different morphological and physiological traits under salinity stress.

Cluster	Germplasm Accessions
1 (Tolerant)	PI518665, PI70023, PI438047, PI438471 (Fiskeby III), PI398847, PI424219A, PI437109C, PI438508, PI506848, PI506849, PI438345, PI561289, PI507692A, PI567201A, PI567568A, PI639571A, PI561365, PI123439, PI170888, PI201428, PI201431, PI597474, PI171442, PI324067, PI430737, PI398285, PI417575, PI346309, PI507433, PI639576, PI639573, PI615451A, PI628803, PI639574, PI628854, PI628809, PI567629B, PI567379B, PI561390, PI628821, PI339982, PI561363, PI628840, PI567521, PI639575, PI605779D, PI578328B, PI561378, PI628887, PI628826, PI639569, PI628814, PI628874, PI578335B, PI605855, PI628819, PI468967, PI567779C, PI567373B, PI561395, PI507417, PI381683, PI468399A, PI407035, PI406684, PI597462C, PI508060, PI507584, PI548656 (Lee)
2 (Moderately Tolerant)	PI533655, PI548986, PI533604, PI620883, PI543832, PI543794, FC31697, PI593463, PI593258, PI615695, PI560909, PI556931, PI170380, PI87530, PI323555, PI92463, PI424149, PI495020, PI210352, PI408100A, PI438343, PI612609, PI567657, PI628828, PI561287B, PI424041, PI407174, PI407065, PI424008C, PI407189, PI407088, PI597460B, PI507619A, PI507581, PI507594A, PI479747, PI483466, PI464938, PI424092, PI424061B, PI378688, PI507795, PI518281, PI507644, PI549046, PI407121, PI407301, PI407316A, PI424026, PI339871C, PI507588, PI597471D, PI522211C, PI424084, PI378686B, PI366119, PI468916, PI483464A, PI424068, PI562565, PI507665, PI507632, PI483468B, PI483462B, PI514674, PI447004, PI597472, PI424072B, PI424017A, PI518282, PI507629, PI507651, PI407029
3 (Susceptible)	PI532833, PI408169D, PI437393, PI424549A, PI437500C, PI438315, PI437550C, PI424364A, PI424285B, PI437392, PI438409, PI438427, PI458065A, PI506850, PI506852, PI506853, PI507676, PI507692B, PI506866, PI438346, PI506863, PI561292B, PI639561, PI567201B, PI506864B, PI572265D, PI153682, PI639571B, PI255734, PI567201D, PI504487, PI561364, PI506865, PI594020, PI639563, PI123577A, PI171430, PI567202, PI639566D, PI36906, PI145079, PI215693, PI371610, PI430736, PI438430, PI438431, PI281885, PI567004, PI256376, PI346306, PI567016, PI615497, PI639565, PI654044B, PI628817, PI628882, PI632942A, PI587612A, PI587846B, PI628883, PI567060A, PI572265C, PI567023A, PI566984, PI567059, PI605756, PI628877, PI615518, PI632936, PI566992B, PI424131, PI518664 (Hutcheson)
4 (Highly Susceptible)	PI602059, PI518669, PI508083, PI510675, PI513382, PI559371, PI534647, PI593259, PI509044, PI525454, PI601984, PI591484, PI633729, PI548657 (Jackson), PI572239, PI542042, PI561400, PI548487, PI85469, PI91083, PI253653A, PI641937, PI84742, PI84671, PI92618, PI253655, PI88287, PI171652, PI253659, PI398752, PI398505, PI398388, PI339865A, PI248402, PI399010, PI361063, PI424159B, PI417573, PI89009.2, PI253652A, PI432359, PI427136, PI437103, PI407812, PI398200, PI253665B, PI424520, PI398920, PI438499, PI437110B, PI83942, PI471934, PI567076, PI572265B, PI468400A

Table S3. Details of SNP makers and linkage disequilibrium in diverse soybean germplasm panel

Chr[a]	Number of SNPs	SNP Start Position[b]	SNP End Position[c]	Chr Length	SNP Density (Kb/SNP)	Average r^2 per chromosome[d]
1	1376	24,952	56,793,551	56,831,625	41.30	0.167
2	1924	51,236	48,566,582	48,577,506	25.25	0.156
3	1390	19,199	45,712,413	45,779,782	32.94	0.143
4	1607	56,409	52,360,037	52,389,147	32.60	0.170
5	1580	12,018	42,194,057	42,234,499	26.73	0.183
6	1591	27,936	51,315,634	51,416,487	32.32	0.170
7	1645	40,207	44,608,799	44,360,647	26.97	0.163
8	1882	117,478	47,796,376	47,837,941	25.42	0.177
9	1558	106,196	50,149,215	50,189,765	32.21	0.159
10	1712	10,780	51,546,040	51,566,899	30.12	0.180
11	1274	17,705	34,706,421	34,766,868	27.29	0.180
12	1369	18,463	40,077,424	40,091,315	29.29	0.197
13	2112	27,757	45,789,704	45,874,163	21.72	0.167
14	1475	38,001	48,997,963	49,042,193	33.25	0.175
15	1924	11,691	51,668,449	51,756,344	26.90	0.152
16	1426	13,090	37,879,369	37,877,015	26.56	0.151
17	1577	60,566	41,616,549	41,641,367	26.41	0.173
18	2408	1,369	57,968,596	58,018,743	24.09	0.158
19	1721	34,329	50,730,824	50,746,917	29.49	0.184
20	1281	26,325	47,895,551	47,904,182	37.40	0.182

[a]Chr: Chromosome
[b]MB position of the first SNP on the chromosome
[c]MB position of the last SNP on the chromosome
[d]r^2 quantifies the strength of linkage disequilibrium

Table S6. Details of the candidate gene primers used for expression analysis in three different soybean genotypes at V2 stage.

Gene ID	Gene symbol	Orien[a]	Sequence	Length	Tm[b]	Function
Glyma.08G194000	*CBL10*	FP	TTGGCACTGTTGAAGACCAC	20	60	CALCINEURIN B-LIKE PROTEIN 10
		RP	TGAGAGCATGCACAAACTCC	20	60	
Glyma.05G007100	*CA1*	FP	ATTGGACACAGTGCTTGTGG	20	60	CARBONIC ANHYDRASE 2, CHLOROPLASTIC-RELATED
		RP	TGCTGTGTCTTCACCTTTGC	20	60	
Glyma.14G200100	*CHX15*	FP	ATACGCAAGACACCCAAAGG	20	60	SODIUM/HYDROGEN EXCHANGER FAMILY
		RP	GCACTGCTAAGCCCAAAATC	20	60	
Glyma.02G127500	*GS2*	FP	TTATTGCAGTGCTGGGACAG	20	60	GLUTAMINE SYNTHETASE
		RP	TGAAGCCCAGATATGATCACC	21	60	
Glyma.01G031800	*KUP6*	FP	TGTTTTCTCGGCTGTGTCTG	20	60	POTASSIUM TRANSPORTER 6
		RP	GGAGCAAACAAGAAGCCAAC	20	60	
Glyma.01G132500	*LTP4*	FP	ATTGCATCGGTTACCTCCAG	20	60	LTP family
		RP	TTGAAGCCGGAGATTTGG	18	60	
Glyma.17G153300	*NRT1.5*	FP	GCATCAAGGAAATGGAGAGC	20	60	PROTEIN NRT1.5
		RP	AACTTGAACCCTTCGGTGTG	20	60	
Glyma.13G307000	*RCI3*	FP	GCTAACAGTTGCCCAAAAGC	20	60	PEROXIDASE 3-RELATED
		RP	CAGCCTGATTGGTTGTTGAG	20	59	
Glyma.08G194100	*PLDEPSILON*	FP	CACACAGAAATCCCACATGC	20	60	PHOSPHOLIPASE D EPSILON
		RP	TCCCAAAGCATAACCCTCAC	20	60	
Glyma.02G073600	*PIP2B*	FP	GACATTTGGGCTCTTCTTGG	20	60	AQUAPORIN PIP2-1-RELATED
		RP	TGCCTTAACCAACCCAACTC	20	60	
Glyma.14G200200	*WRKY33*	FP	TCTGCCATCTCCAACAACTG	20	60	WRKY DNA-binding protein 33
		RP	CTGATCGGGTTTGGAAAGAG	20	60	
Glyma.03G171600	*GmSALT3*	FP	ACCACGCTCTTCCTTTGTTG	20	60	CATION/H(+) ANTIPORTER 20
		RP	TGACGAAAGGGTTTGAGGAG	20	60	
Glyma.02G072800	*RAP2.11*	FP	TGTGGCTTGGCACATATGAG	20	60	RAP2.11
		RP	TGAAGTTGGTGCGAGTGTTG	20	60	
Glyma.20G138400	*CRK29*	FP	ACAAGGTGGATTTGGAGCTG	20	60	Cysteine-rich RLK (RECEPTOR-like protein kinase) 29
		RP	AGCTTGGCCACTAAAAGCAC	20	60	
Glyma.02G091900	*GmActin*	FP	CCGGTCGTGACCTCACTGATTTCT	24	60	GmActin
		RP	CATCAGGCAACTCGTAGCTCTTCTCG	26	60	
Glyma.02G276600	*GmEF1A*	FP	CCACTGCTGAAGAAGATGATGATG	24	60	Elongation Factor 1
		RP	AAGGACAGAAGACTTGCCACTC	22	60	

[a]Orien, Orientation of primers: FP, Forward primer; RP, Reverse primer; [b]Tm, melting temperature

Table S7. Testing of the association of *GmSALT3 (Glyma03g32900*) with salt tolerance using the markers flanking the gene. This was done by testing the allelic effects of markers flanking the gene in the germplasm accession used in the study.

Trait[a]	SNP Surrounding *GmSALT3*	Chr[b]	Position	P value	MAF[c]	Marker Effect
LSS	ss715585949 (Left Marker)	3	38601323	0.40	0.44	-0.04
	ss715585952 (Right Marker)	3	38644367	0.78	0.34	0.01
CC	ss715585949 (Left Marker)	3	38601323	0.44	0.44	0.19
	ss715585952 (Right Marker)	3	38644367	0.53	0.34	-0.15
LCaC	ss715585949 (Left Marker)	3	38601323	0.62	0.44	-0.06
	ss715585952 (Right Marker)	3	38644367	0.71	0.34	-0.05
LPC	ss715585949 (Left Marker)	3	38601323	0.05	0.44	0.44
	ss715585952 (Right Marker)	3	38644367	0.56	0.34	0.34
LSC	ss715585949 (Left Marker)	3	38601323	0.54	0.44	-0.33
	ss715585952 (Right Marker)	3	38644367	0.74	0.34	0.18
LCC	ss715585949 (Left Marker)	3	38601323	0.35	0.44	-0.10
	ss715585952 (Right Marker)	3	38644367	0.32	0.34	0.11
LNaK	ss715585949 (Left Marker)	3	38601323	0.51	0.44	0.01
	ss715585952 (Right Marker)	3	38644367	0.34	0.34	-0.01

[a]LSS, leaf scorching score; CC, chlorophyll content; LCaC, leaf calcium content; LPC, leaf potassium content; LSC, leaf sodium content; LCC, leaf chloride content; LNaK; leaf sodium to potassium ratio

[b]Chr, Chromosome; [c]MAF, minor allele frequency
